# SUMO monoclonal antibodies vary in sensitivity, specificity, and ability to detect types of SUMO conjugate

**DOI:** 10.1038/s41598-022-25665-6

**Published:** 2022-12-09

**Authors:** Alexander J. Garvin, Alexander J. Lanz, Joanna R. Morris

**Affiliations:** Birmingham Centre for Genome Biology and Institute of Cancer and Genomic Sciences, College of Medical and Dental Schools, University of Birmingham, Birmingham, B15 2TT UK

**Keywords:** Enzymes, Immunochemistry, Proteins, Immunoblotting, Immunoprecipitation

## Abstract

Monoclonal antibodies (MAb) to members of the Small Ubiquitin-like modifier (SUMO) family are essential tools in the study of cellular SUMOylation. However, many anti-SUMO MAbs are poorly validated, and antibody matching to detection format is without an evidence base. Here we test the specificity and sensitivity of twenty-four anti-SUMO MAbs towards monomeric and polymeric SUMO1-4 in dot-blots, immunoblots, immunofluorescence and immunoprecipitation. We find substantial variability between SUMO MAbs for different conjugation states, for detecting increased SUMOylation in response to thirteen different stress agents, and as enrichment reagents for SUMOylated RanGAP1 or KAP1. All four anti-SUMO4 monoclonal antibodies tested cross-reacted wit SUMO2/3, and several SUMO2/3 monoclonal antibodies cross-reacted with SUMO4. These data characterize the specificity of twenty-four anti-SUMO antibodies across commonly used assays, creating an enabling resource for the SUMO research community.

## Introduction

The SUMO family consists of three conjugated members (SUMO1-3), a non-conjugatable SUMO4^1^ and SUMO5/SUMO1P1, which has restricted tissue expression^2^. SUMO1-3 are processed into mature, conjugatable forms through the removal of the extreme C-terminal residues^3^. SUMO1 and SUMO2/3 use the same conjugation machinery^4,5^, and SUMO proteins can be conjugated as monomers, multi-monomers and polymers. They can form multiple internal lysine linkage types, including branching and mixed chains comprised of different SUMO family members and other Ub/Ubls^6^. Conjugation of SUMO (SUMOylation) is essential for several cellular processes, including transcription, DNA replication, mitosis, genome stability and immunity^7–12^. Transient up-regulation of SUMOylation is associated with responses to cellular stress^13^. SUMOylation can alter protein localization, activity, turnover, and protein interactions^14,15^. SUMOylation is a transient process often confined to a subset of the target protein that may be spatially and temporally restricted. SUMO proteases (SENP1-7), USPL1 and DeSi1/2 deconjugate SUMO from substrates contributing to the balance of SUMOylation and deSUMOylation^16–21^.

Advances in proteomic analysis of SUMO conjugation have enhanced the cataloguing of the global SUMOylome^22–24^ with further adaptions reducing dependency on over-expressed epitope-tagged SUMO^25,26^. Enrichment with SUMO interacting trap proteins^27,28^ or BioID^29^ have further enhanced our understanding of the SUMOylated proteome. Detection of SUMO conjugation is challenging due to the small proportion of modified substrate, its transient, often context-dependent nature and the rapid deconjugation by SENP enzymes. Thus, in large part, SUMOylation studies rely on detecting endogenous SUMO family members using antibodies.

Approximately one hundred SUMO1-4 monoclonal antibodies (MAbs) are commercially available (Supplemental Table 1). Of these, a minority are cited (Supplemental Fig. 1a), and most are incompletely or not validated by their vendors (Supplemental Fig. 1b,c). Poor antibody characterization is a contributor to the reproducibility crisis in research^30,31^. Indeed, a systematic attempt to validate seven reported neuronal SUMO1 conjugated proteins using an HA-SUMO1 knock-in mouse failed to confirm SUMO1 conjugation for any of the substrates^32,33^. While differences in methodology, expression levels and animal models may explain some of these issues, significant deficiencies in available SUMO1 antibodies contributed to reproducibility difficulties^34,35^. Additionally, our anecdotal experience has shown anti-SUMO antibody variability when detecting SUMO conjugation after ionizing radiation treatment^36^. Here we catalogue the specificity and sensitivity of SUMO MAbs to encourage reproducibility within SUMO biology studies and highlight their strengths and weaknesses.

## Results

We chose twenty-four MAbs from the ninety-three SUMO1-4 MAbs commercially available at the time of writing (Supplemental Table 1); nine were raised against SUMO1, eleven against SUMO2/3 and four against SUMO4. They were selected based on high citations from the CiteAb database as a proxy for research community usage, and each had validation data available on the manufacturer's websites^37^. Antibodies were raised in mice, rabbits and rats and used a variety of immunogens, including recombinant GST-SUMO, untagged SUMO, and peptides. With two exceptions (8A2 and 21C7), the antibody epitopes had not been mapped, or the identity of the peptide immunogen was proprietary (Supplemental Table 1).

In the current study, antibodies were tested at 1 µg/mL except for recombinant antibodies (EPR300, EPR4602, EPR7163, JJ-085 and ARC1382) or antibodies from Cell Signalling Technologies (C9H1 and 18H8), which are supplied at lot-specific dilutions. In these exceptions, antibodies were diluted at 1:1000 in 5% milk. As some of the MAbs used are available from multiple vendors, they are referred to by clone name rather than catalogue number throughout.

### Sensitivities and specificity of MAbs for monomeric SUMO

**Figure 1:**
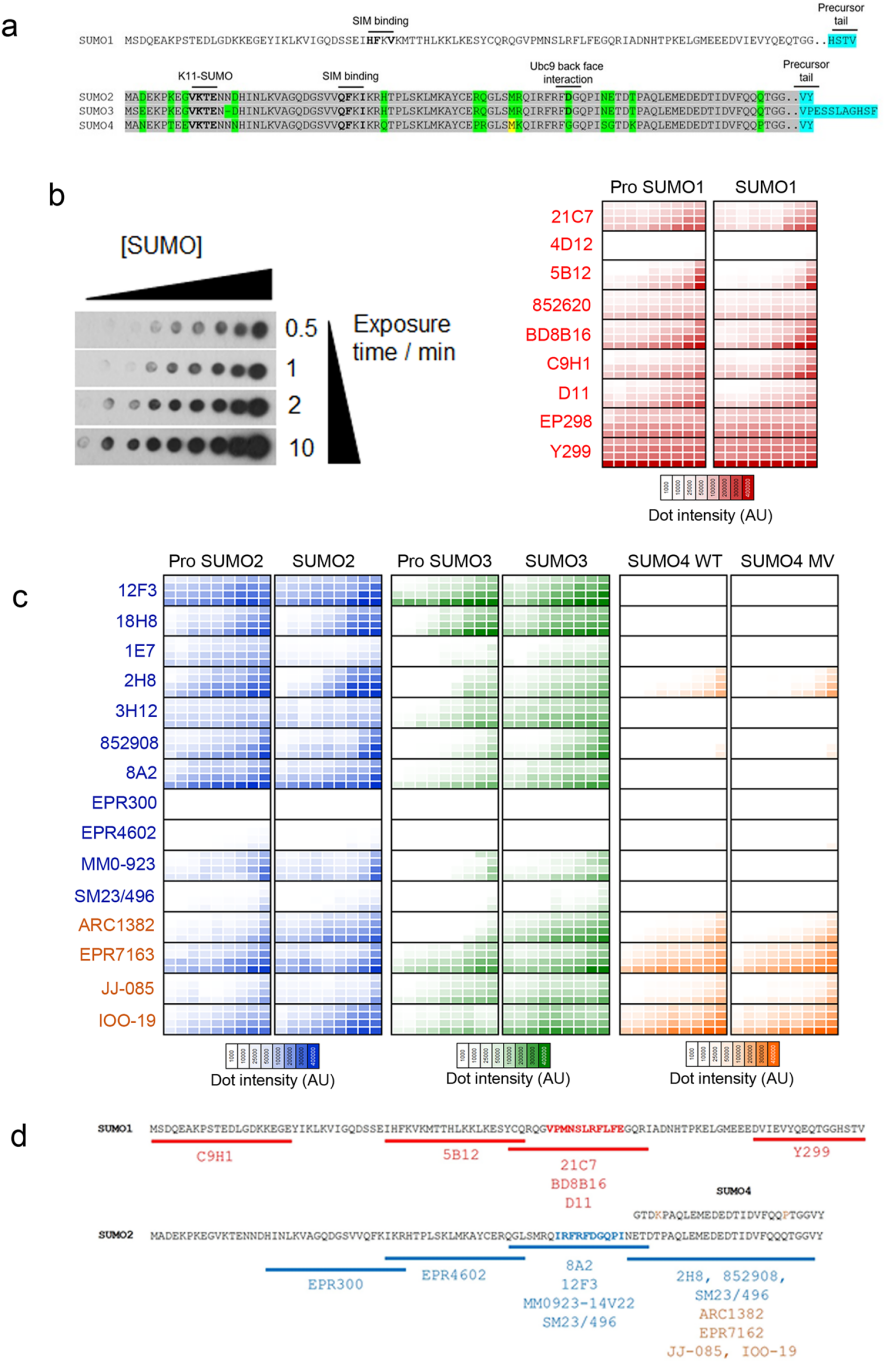
Variable sensitivity and selectivity of SUMO MAbs to detect monomeric SUMO. **(a)** Illustration of SUMO1-4 sequence. Amino acid sequences of human SUMO1 (P63165), SUMO2 (P61956), SUMO3 (P55854) and SUMO4 (Q6EEV6). The major SUMO acceptor K11, SIM (SUMO Interacting Motif) contacting residues, Ubc9 back face interacting residues are highlighted in bold. Precursor tail residues cleaved by SENP enzymes are indicated in blue. Conserved residues between SUMO2-4 are highlighted in grey, while non-conserved residues are highlighted in green. The variable M55 residue in SUMO4 is highlighted in yellow. (**b)** Right—heat map of SUMO1 MAbs detecting pro and mature rSUMO1. rSUMO1 was spotted (1 μL) at a concentration of 3 μg/μL with half dilutions per spot down to 3 ng/μL. A deeper colour signifies a more intense chemiluminescent signal, for four exposure time points (0.5, 1, 2 and 10 min—shown vertically) as a mean of three independent repeats based on densitometry of chemiluminescent detection on X-ray film. Left -a representative dot blot is shown with increasing concentration of rSUMO1 on the horizontal and increasing film exposure time on the vertical. N = 3. (**c)** Heat map of SUMO2/3/4 MAbs detecting pro and mature rSUMO2/3/4. As for 1b, in this case, each MAb was tested against SUMO2 and SUMO3 (Pro and mature forms) and SUMO4 (WT and M55V variant). SUMO2/3/4 were diluted at the same concentrations as rSUMO1. SUMO2/3 MAbs are in blue text and SUMO4 MAbs are in orange. N = 3. (**d)** Epitope mapping of SUMO MAbs. Partially overlapping SUMO1, SUMO2 or SUMO4 peptides, shown by underline, were used for competition assays. Loss of detection (by dot blot or immunoblot of SUMO conjugates) was interpreted as indicating the location of individual MAb epitopes. The SUMO1 region highlighted in red shows the mapped 21C7 epitope. The SUMO2 region highlighted in blue shows the 8A2 epitope. SUMO4 MAbs are shown in orange. The sequence of SUMO4 is shown above, with the two non-conserved residues highlighted in orange. MAbs not shown could not be mapped.

To test the sensitivity and specificity of the antibodies, we generated recombinant SUMO1-4 purified from *E. coli* (rSUMO1-4). For SUMO1-3, we generated both immature/ProSUMO (containing an extended C-terminal sequence, Fig. 1a) and mature (terminating in GG) forms as some of the antibodies were generated against ProSUMO (Supplemental Table 1). SUMO4 is not processed into a mature form^38^. For this protein, we purified WT SUMO4 and the M55V variant (rs237025). The polymorphism underlying SUMO4 M55V is prevalent and associated with several human pathologies, including diabetes^39^.

Each SUMO1 antibody was tested by dot blot of serially diluted rProSUMO1 or rSUMO1. As SUMO1 shares just 50% amino acid identity with SUMO2-4, we did not test cross-reactivity (Fig. 1a). Dot blots were exposed to film for four time points (0.5, 1, 2 and 10 min). A representative dot blot for rSUMO1 is shown in Fig. 1b. For comparison between MAbs, the chemiluminescent intensity of each spot is shown as a heat map with deeper red indicating higher chemiluminescent intensity. A range of sensitivities was clear. 4D12 showed almost no signal, while Y299 produced a saturating chemiluminescent signal even with a low concentration of rSUMO1 (Fig. 1b). We detected no differences in the ability of the MAbs to detect pro-SUMO1 *Vs* mature forms of rSUMO1 (Fig. 1b). Antibodies raised against SUMO2/3 (highlighted in blue) also showed a range of sensitivities to rSUMO2-4, from almost no signal (EPR4602, EPR300, SM23/496) to a strong, specific signal (8A2 and 12F3) with no differences in the ability to detect pro and mature SUMO2/3 forms (Fig. 1c). No antibody showed a preference for SUMO2 or SUMO3. The SUMO2/3 MAbs 2H8, and to a lesser degree 852908, detected rSUMO4 by dot blot. The four antibodies raised to SUMO4 (highlighted in orange) detected rSUMO4 to varying degrees, with none showing differential detection of the M55V variant. All showed cross-reaction to SUMO2/3 proteins (Fig. 1c).

We then used peptide competition assays to understand antibody cross-reactivity better. We confirmed the location of 21C7 and 8A2 epitopes, which had previously been mapped^25,40^ and provided approximate epitope locations for 18/24 MAbs (Fig. 1d). All four antibodies raised against SUMO4 detected the C terminus of SUMO4, a region of high (92%) conservation with SUMO2/3 (Fig. 1a), and the two most SUMO4 cross-reactive SUMO2/3 clones (2H8 and 852908) also recognized this region.

Overall, we find a wide range of sensitivities for monomeric SUMO, little specificity for pro *Vs* mature SUMO, and the antibodies raised to SUMO4 cross-react with SUMO2/3.

### Varied sensitivity and specificity for SUMO family members in cell lysates

**Figure 2:**
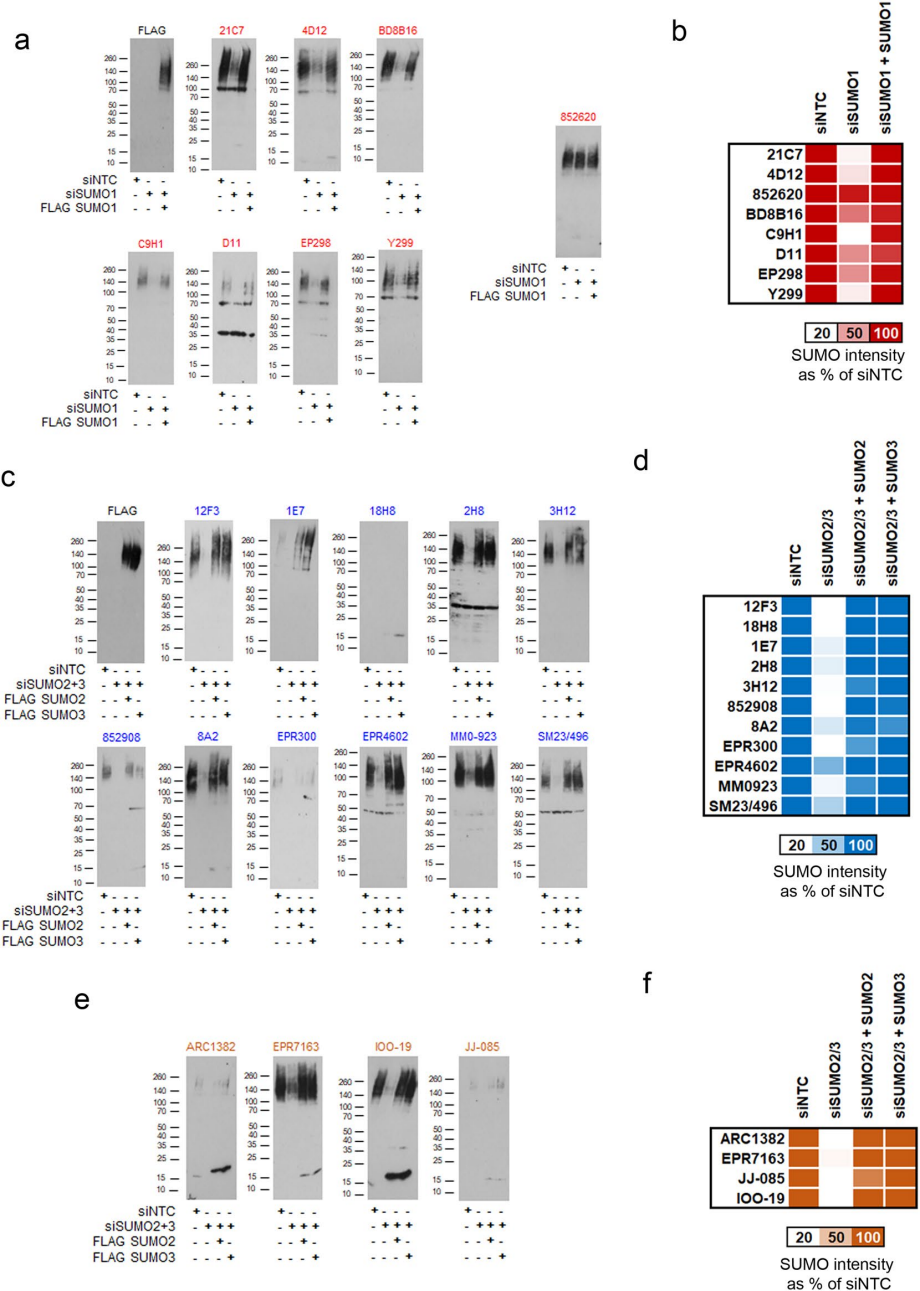
Specificity of SUMO MAbs by immunoblot. **(a)** U2OS cells with the ability to express siRNA-resistant FLAG-SUMO1 treated with non-targeting control siRNA (siNTC), or siRNA to SUMO1 (siSUMO1) or treated with siSUMO1 and with doxycycline for 48 h to induce FLAG-SUMO1. Lysates were separated by 4–20% SDS-PAGE and immunoblotted with indicated MAbs. All blots are shown at 1 min exposure. (**b)** Heat map of total SUMO1 signal (10 kDa to well front) expressed as a % relative to siNTC. Deep red indicates high rescue of the siRNA-resistant FLAG-SUMO1. N = 3. (**c)** U2OS cells able to express siRNA-resistant forms of FLAG-SUMO2 and FLAG-SUMO3 were treated with siRNA to both SUMO2 and SUMO3 and probed with the SUMO2/3 antibodies shown. (**d)** Heat map relative quantification of SUMO2/3 signal (10 kDa to well front) detected by antibodies raised to SUMO2/3. N = 3. (**e)** Lysates from 2c probed with SUMO4 MAbs. (**f)** Heat map relative quantification of the SUMO2/3 signal (10 kDa to well front) detected with SUMO4 Mabs. N = 3.

Next, we sought to determine specificity and selectivity against cellular SUMO conjugates. We generated cells with inducible cDNAs for SUMO1, SUMO3 and SUMO4 containing synonymous mutations rendering them insensitive to siRNA depleting the endogenous SUMO transcripts (Supplemental Fig. 2a). Endogenous SUMO2 was targeted using 3' UTR siRNA (Supplemental Fig. 2a). Simultaneous siRNA depletion and doxycycline addition allowed a degree of replacement of endogenous SUMO with FLAG-tagged variants that could be probed with the well-established anti-FLAG antibody, M2, as a comparison to SUMO MAbs (Fig. 2).

Western blots of siRNA-depleted and FLAG-SUMO complemented U2OS lysates confirmed the specificity of several SUMO1 MAbs (21C7, 4D12, C9H1 and Y299). BD8B16 was intermediate in specificity. The intensity of the SUMO1 conjugates was converted into a heat map (Fig. 2b) to summarise the blots (Fig. 2a). Signals detected by D11 and EP298 showed some reduction following siRNA treatment but also detected non-specific bands unaffected by siSUMO1 treatment (Fig. 2a,b). Fractionation of cell lysates indicated these non-specific bands arise from cytoplasmic proteins and not from chromatin, where most SUMO1 conjugates are found in interphase cells (Supplemental Fig. 2b). MAb 852620 detected proteins that were unchanged by SUMO1 siRNA treatment. The conjugate banding pattern detected by this clone was also unchanged when cells were treated with SUMO1 E1 inhibitor ML-792^41^, the NEDD8 E1 inhibitor MLN4924 and were not increased by the ubiquitin E1 inhibitor TAK-243, which has been reported to increase SUMOylation^42^ (Supplemental Fig. 2c). Therefore, the signal detected by 852620 is unlikely to belong to ubiquitin, NEDD8 or SUMO conjugates. Clone 5B12 failed to detect SUMO1 conjugates in multiple conditions and was not tested further by immunoblot. Representative 5B12 blots, related to Figs. 2a and 4b are shown in the final section of uncropped blots in the supplemental information section.

SUMO2/3/4 MAbs were tested in a similar fashion, instead using combined siRNA treatment to SUMO2 and SUMO3 in FLAG-SUMO2 or FLAG-SUMO3 complemented cell lines. Antibodies raised to SUMO2/3 showed reduced immunoblot signal when probing lysates from SUMO2/3 siRNA-depleted cells and showed similar detection of FLAG-SUMO2 and FLAG-SUMO3 (Fig. 2c,d). Therefore, the majority of SUMO2/3 MAbs specifically detect SUMO2/3 conjugates. All four SUMO4 MAbs showed cross-reaction with SUMO2/3 conjugates (Fig. 2e,f). To further assess cross-reaction between SUMO2/3 and SUMO4 MAbs we over-expressed FLAG-HA SUMO4 in U2OS and tested the resulting lysates (Supplemental Fig. 2d–h). Consistent with the ability of SUMO2/3 MAbs to detect SUMO2/3 conjugates—high molecular weight conjugates were detected by SUMO2/3/4 MAbs; additionally, several SUMO2/3 MAbs detected the monomeric FLAG-HA SUMO4 (Supplemental Fig. 2h). Consistent with the dot blot analysis (Fig. 1c), two SUMO2/3 MAbs—2H8 and 852908 showed similar sensitivity to FLAG-HA SUMO4 as the four SUMO4 MAbs, further confirming these antibodies can detect SUMO2/3/4. Thus, while sensitive for SUMO2/3 high molecular weight conjugates, several SUMO2/3 MAbs can also detect exogenous unconjugated SUMO4, likely due to the high degree of conservation between the SUMO2/3/4 family members (Fig. 1a).

### Differences in the ability to detect free and conjugated SUMO

**Figure 3:**
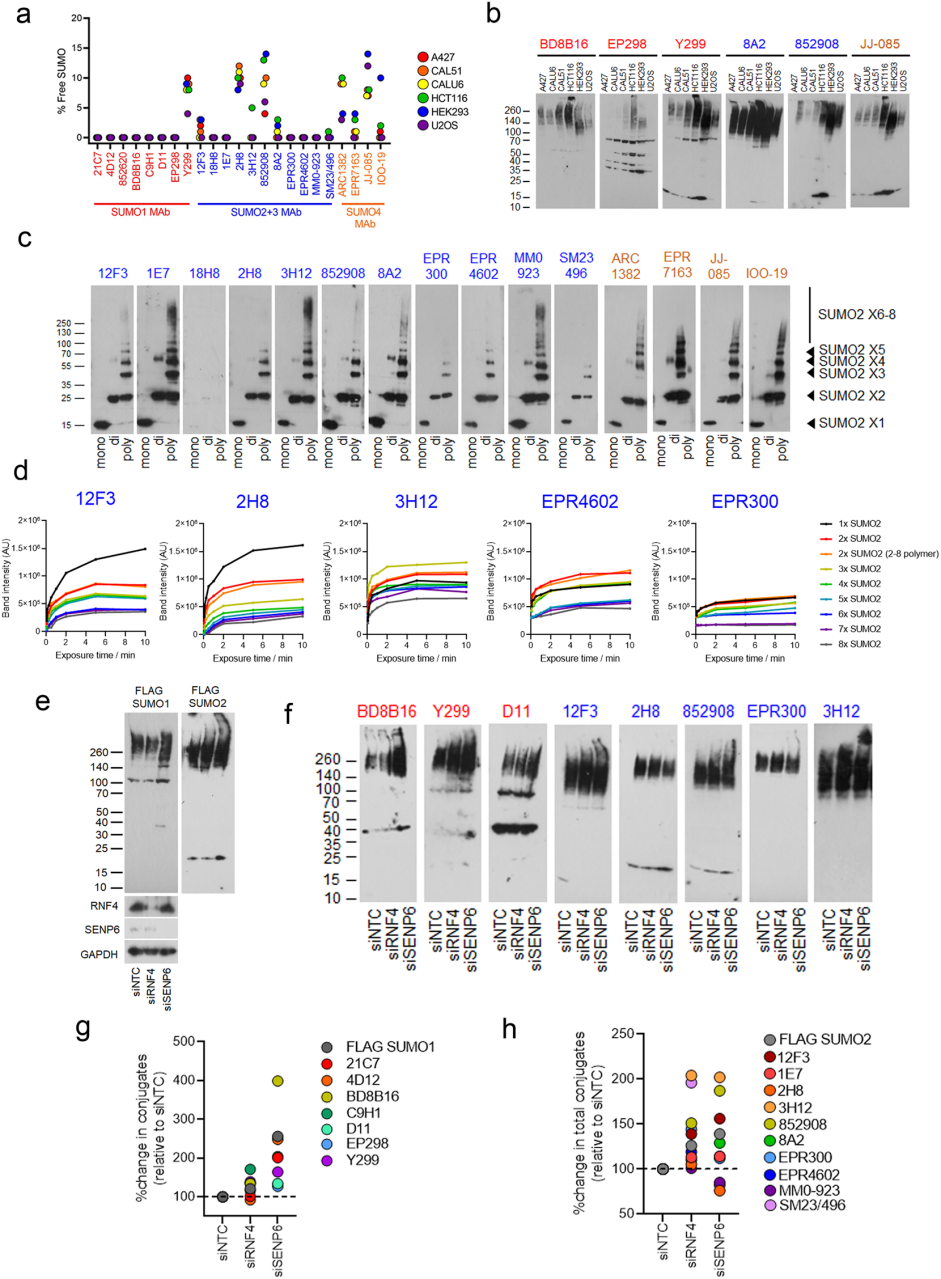
SUMO Mabs vary in ability to detect SUMO conjugate types. **(a)** The % ~ 15 kDa band (interpreted as free SUMO protein) as a proportion of the total SUMO signal is shown for each of six cell lines, across all MAbs. The % free SUMO is calculated as free SUMO/total SUMO (conjugates and free) × 100 (blots from multiple exposures were quantified and averaged to ensure the linearity of the signal). N = 3. (**b)** Representative blots of lysates from indicated cell lines, the band at ~ 15 kDa is interpreted as free SUMO. Images for all cell lines and exposures can be found in supplemental Fig. 3A. (**c)** Immunoblots of SUMO2 monomers, dimers, and polymers (2–8 × SUMO2) at 200 ng/lane resolved on 4–20% SDS PAGE gels. Images are from various film exposure times. All exposures can be found in Supplemental Fig. 3b. (**d)** Quantification of representative experiments from (**c)** from multiple exposure times. (**e)** Immunoblots of U2OS FLAG-SUMO1 or FLAG-SUMO2 cells, siRNA treated as indicated and doxycycline-treated for 48 h to replace endogenous SUMO with FLAG-SUMO. Immunoblots of RNF4 and SENP6 siRNAs are shown. (**f**) Representative immunoblots FLAG-SUMO complemented U2OS lysates from (**e**) probed with representative SUMO1 MAbs (red) and SUMO2/3 MAbs (blue). Blots for all MAbs are shown in the uncropped membrane section in the supplemental data. (**g)** The % change in the total signal detected by antibodies raised to SUMO1 following siRNA depletion of RNF4 and SENP6 relative to non-targeting (NTC) siRNA control in the cell lines shown N = 3. (**h)** The % change in the total signal detected by antibodies raised to SUMO2/3 following siRNA depletion of RNF4 and SENP6 relative to NTC siRNA control in the cell lines shown N = 3.

We next quantified the % of free SUMO (~ 15 kDa band) detected by each antibody in lysates from a series of cell lines (A427, CAL51, CALU6, HCT116, HEK293 and U2OS). The majority of Mabs did not detect a signal consistent with free SUMO protein in immunoblots, for the remainder MAbs (Y299, 12F3, 2H8, 852908, 8A2, ARC1382, EPR7163, JJ-085 and IOO-19) the free SUMO band accounted for 10–20% of the total signal (Fig. 3a,b and Supplemental Fig. 3a). Next, we tested the monomeric, dimeric and polySUMO2 protein detection by antibodies raised to SUMO2/3/4 and quantified band intensities at increasing film exposure times (Fig. 3c,d and Supplemental Fig. 3b). The experiment revealed that some MAbs preferred monomeric SUMO2 over polymeric forms (12F3, 2H8, 852908, 8A2 and SM23/496); others showed less preference (1E7, 3H12, EPR4602, MM093-14V22). Some poorly detected high molecular weight SUMO2 conjugates (18H8 and EPR300). All four antibodies raised to SUMO4 bound to SUMO2 monomers, dimers, and polymers, confirming their cross-reaction with SUMO2 (Fig. 3c).

To further explore the potential for each MAb in the analysis of cellular conjugates, we increased SUMO polymer levels by depleting the polySUMO-targeted ubiquitin ligase RNF4 and polySUMO-specific protease SENP6 in FLAG-SUMO1 or FLAG-SUMO2 complemented cells. For SUMO1 MAbs, siRNF4 promoted insignificant alterations in conjugates as detected by FLAG M2, while C9H1 detected more pronounced changes (Fig. 3e–g and uncropped blots in supplemental information). Depletion of SENP6 increased FLAG-SUMO1 conjugates more profoundly, and SUMO1 MAbs showed a wide spread of conjugate detection from a ~ 30% increase (D11 and EP298) to a > 200% increase (21C7, 4D12 and BD8B16) (Fig. 3e–g). These observations suggest SUMO1 Mabs can differentially detect SUMO1 conjugates antagonized by SENP6. FLAG-SUMO2 conjugates were increased by both siRNF4 and siSENP6 (Fig. 3e–g). The detection of SUMO2 conjugates also varied widely between SUMO2/3 MAbs. The clone 3H12 detected a > 200% increase in SUMO2 conjugates in RNF4 and SENP6 depleted lysates, notably the very high molecular weight (> 260 kDa) SUMO species indicative of extensively modified conjugates (Fig. 3f), consistent with 3H12 strongly detecting higher molecular weight SUMO2 polymers in Fig. 3c,d. Conversely, the SUMO2/3/4 MAb 2H8 detected modest increases in FLAG-SUMO2 conjugates on RNF4 depletion and a small decrease in SENP6 siRNA depletions (Fig. 3f,h), consistent with a preference for monoSUMO2/3/4 detection (Fig. 1c), strong detection of free SUMO (Fig. 2a) and poor recognition of longer SUMO2 polymers (Fig. 3c,d). Collectively these data show SUMO MAbs vary substantially in their ability to distinguish different forms of SUMO conjugation.

### Detection of SUMO conjugates on stress

**Figure 4:**
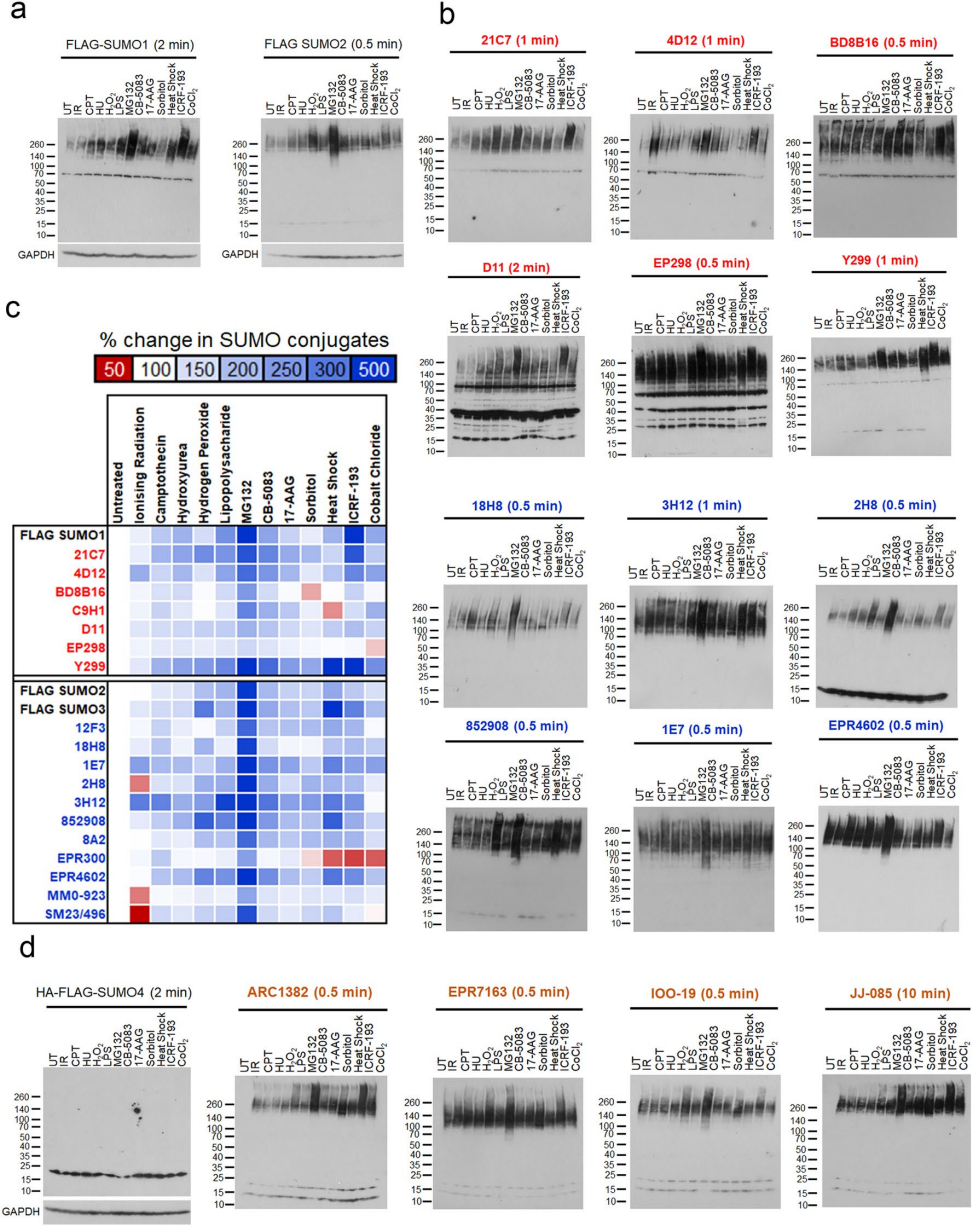
Detection of changes in SUMO conjugate states by cellular stress inducers. **(a)** U2OS cells treated with siRNA targeting endogenous SUMO1 or SUMO2/3 and doxycycline-treated to induce siRNA-resistant FLAG-SUMO1 or siRNA-resistant FLAG-SUMO2 for 48 h before treatment with indicated stress agents. UT (Untreated control), IR (Ionising Radiation, 4 Gy, 1 h post), CPT (Camptothecin 1 µM, 3 h), HU (Hydroxyurea 5 mM, 3 h), H_2_O_2_ (Hydrogen Peroxide 125 µM, 15 min), LPS (Lipopolysaccharide 1000 ng, 3 h), MG132 (5 µg/mL, 3 h), CB-5083 (0.1 µM, 3 h), 17-AAG (5 µM, 3 h), Sorbitol (500 mM, 30 min), heat shock (42 °C, 10 min), ICRF-193 (100 µM, 1 h). Immunoblots were probed with anti-FLAG antibody. Exposure time is shown, all exposures are shown in Supplemental Fig. 4a,b. (**b)** Representative immunoblots of lysates (**a**) probed with MAbs raised against SUMO1 in red (21C7, 4D12, BD8B16, D11, EP298 and Y299) and SUMO2/3 in blue (18H8, 3H12, 2H8, 852908, 1E7 and EPR4602) with exposure times shown. All exposures and Mabs tested are shown in Supplemental Fig. 4a,b. (**c)** Heat map relative quantification summarising changes in signal after treatment for each SUMO1 or SUMO2/3 MAb. % Change calculated from three experimental repeats per MAb using densitometry of proteins migrating above 70 kDa as a % change relative to the untreated control lane for each immunoblot. Free SUMO was not included in the quantification. The mean for each condition and MAb is shown. Red indicates a reduction in SUMO conjugates relative to siNTC, white no change and blue an increase. (**d)** U2OS cells treated with siRNA targeting endogenous SUMO4 and expressing siRNA-resistant FLAG-HA SUMO4 and treated as for (**a**). Lysates were then probed with MAbs raised to SUMO4 (orange). All exposures are shown in Supplemental Fig. 4a–c.

SUMOylation is highly responsive to cellular stress, proteomic evidence suggests that different stresses alter both the substrate identities and types of SUMO modifications^26,43^. To assess the ability of SUMO MAbs to detect gross stress-induced changes, we treated FLAG-SUMO1 or FLAG-SUMO2 complemented U2OS with thirteen agents. The agents were chosen to induce genotoxic (IR and CPT), replicative (HU), oxidative (H_2_O_2_), proteotoxic (MG132, CB-5083 and 17-AAG), osmotic (sorbitol), thermal (heat shock) and mitotic (ICRF-193) stresses. The antibody, M2, detecting the tag, FLAG, of the exogenously expressed SUMOs was used to compare changes in FLAG-SUMO1 or FLAG-SUMO2 conjugates (Fig. 4a). The conditions tested resulted in varying degrees of FLAG-SUMO conjugate changes (Fig. 4b), summarised by a heat map of relative changes in SUMO conjugate intensity (Fig. 4c). Treatment with the proteasome inhibitor, MG132, increased the high molecular-weight SUMO signal detected by all tested antibodies (Fig. 4a–d and Supplemental Fig. 4a,b). Three antibodies detected large increases in high molecular-weight conjugates following irradiation (4D12, 1E7 and 3H12); the rest detected small increases, or in some instances (2H8, MM0-923 and SM23/496) detected a decrease in signal from high molecular weight proteins. Similarly, wide-ranging changes in SUMO conjugates were observed after treatment with the oxidative stress inducer hydrogen peroxide, which resulted in an increased conjugate signal detected by FLAG M2, 21C7, Y299 18H8, 1E7, 2H8, 3H12, 852908, 8A2, and EPR4602 (Fig. 4c). In contrast, no substantive change was detected by BD8B16, C9H1, 12F3, EPR300 or MMO-923 clones (Fig. 4c). In lysates of cells treated with the topoisomerase II (Topo-II) catalytic inhibitor ICRF-193, FLAG M2 and clones 21C7 and Y299 detected a large increase in FLAG-SUMO1 conjugates, whereas these were less strongly detected by BD8B16, C9H1 and D11 and barely detected by EP298 clones (Fig. 4b,c). These observations suggest SUMO1 conjugates induced by Topo-II inhibitors are not universally detectable by all SUMO1 MAbs.

SUMO4 MAbs also detected stress-dependent changes in high molecular weight conjugates, consistent with their ability to cross-react with SUMO2/3 conjugates. Free FLAG-HA-SUMO4 detected by M2 MAb showed no conjugate formation on stress (Fig. 4d and Supplemental Fig. 4c). These observations further strengthen the notion that SUMO4 does not form detectable conjugates on stress and that changes in conjugate pattern detected by SUMO4 MAbs more likely correspond to changes in SUMO2/3 conjugate status.

### Specificity of MAbs for indirect immunofluorescence

**Figure 5:**
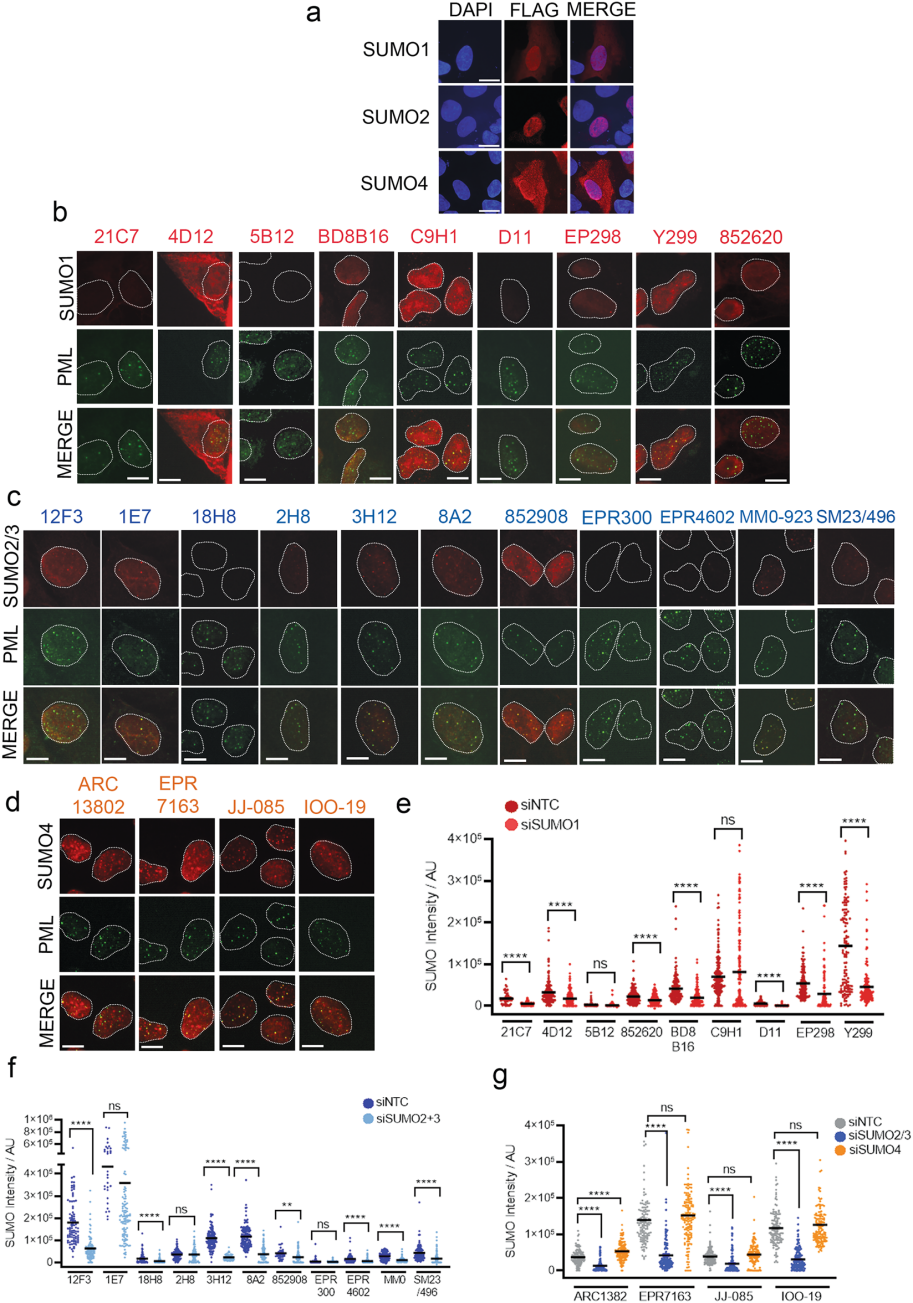
SUMO MAbs as detection tools for subcellar microscopy. **(a)** Detection of exogenous FLAG-SUMO1, FLAG-SUMO2 and FLAG-HA SUMO4 in U2OS by indirect immunofluorescence using anti-FLAG antibodies. Scale bar = 10 µm. (**b)** U2OS cells stained with indicated anti-SUMO1 MAb (red) and PML (green). Mouse and Rat SUMO1 MAbs (21C7, 4D12, 5B12, BD8B16, D11 and 852620) were co-stained with Rabbit PML antibody and Rabbit SUMO1 MAbs were co-stained with Mouse PML antibody. Scale bar = 10 µm. To improve visibility brightness was increased by 20% across all panels. (**c)** Cells stained as for (**b**), using anti-SUMO2/3 MAbs. To improve visibility brightness was increased by 20% across all panels. (**d)** Cells stained as for (**b**) using anti-SUMO4 MAbs. (**e)** Quantification of SUMO1 MAb intensity signal per whole cell treated with either control (siNTC) or SUMO1 (siSUMO1) siRNA for 72 h prior to fixation. Two-tailed t-test denotes statistical differences between siNTC and siSUMO1. N = ~ 100 cells per condition. (**f)** Quantification of the anti-SUMO2/3 MAb signals in the presence and absence of SUMO2 + 3 siRNA. N = ~ 100 cells per condition. (**g)** Quantification of the anti-SUMO4 MAb signals in the presence and absence of SUMO2 + 3 and SUMO4 siRNA. N = ~ 100 cells per condition.

We next tested the ability of the MAb clones to detect the subcellular localization of SUMOs by immunofluorescence in fixed U2OS cells. Using siRNA-complemented U2OS, we found exogenous FLAG-SUMO1 and FLAG-SUMO2 predominantly localized to the nucleus, while FLAG-HA SUMO4 could be found in both the nucleus and cytoplasm (Fig. 5a). The SUMO1 MAbs detected variable localization patterns, with some showing a diffuse nuclear signal (BD8B16, EP298 and 852620), and others a concentration of dot-like structures, which co-localized with PML (C9H1 and Y299). SUMO1 MAb clone 4D12 signal could be detected in both the cytoplasm and nucleus (Fig. 5a,b). The majority of SUMO2/3 MAbs produced a pan-nuclear and dot/PML co-localizing staining pattern (Fig. 5c). MAbs raised to SUMO4 exhibited predominantly nuclear signals more closely resembling SUMO2 than SUMO4 staining patterns (Fig. 5a,d). To test specificity, we immunostained U2OS treated with SUMO siRNAs. Antibody staining signals were reduced by relevant siRNA depletion for some clones (e.g. anti-SUMO1 MAbs: BD8B16, Y299; anti-SUMO2/3 MAbs: 3H1, 8A2); others were not reduced (e.g., anti-SUMO2/3 MAbs: 2H8, EPR300 and 4D12) (Fig. 5e,f). The majority of the signal detected by SUMO4 MAbs was lost following siSUMO2/3-treatment but not following siSUMO4, confirming that these MAbs also cross-react with SUMO2/3 in staining fixed proteins (Fig. 5g). In some cases, staining was affected by the use of pre-extraction prior to fixation—likely reflecting the ability of some SUMO MAbs to detect non-specific cytoplasmic or soluble proteins (Supplemental Fig. 5). Taken together, these data show that the antibodies raised to SUMO vary greatly in their sensitivity and specificity of SUMO protein detection in fixed cells observed by indirect immunofluorescence.

### SUMO MAbs as enrichment agents

A common methodology for detecting SUMO conjugation of individual substrates is to use a SUMO antibody as an enrichment tool followed by immunoblotting against the studied substrate. Given the variability in sensitivity and specificity described herein, we set out to determine the ability of the MAbs to purify known SUMOylated substrates by immunoprecipitation (IP). Using FLAG-SUMO complemented cells, we were able to compare IP efficiency from multiple antibodies in parallel using the anti-FLAG antibody, M2. We used a modified version of the denaturing IP protocol developed by Becker et al*.*, 2013^25^.

**Figure 6:**
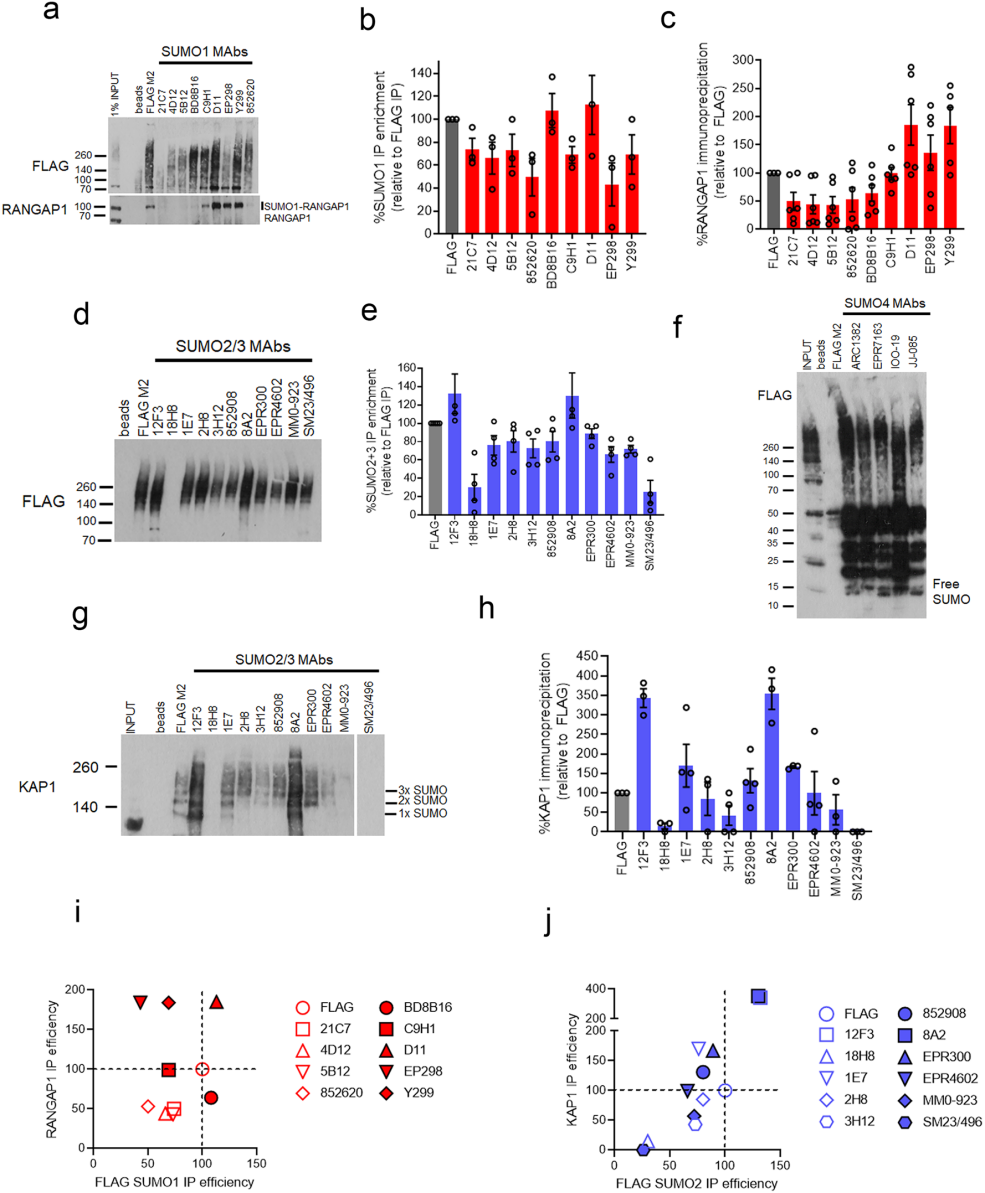
SUMO MAbs as SUMOylation enrichment tools. **(a)** U2OS cells with the ability to express siRNA-resistant FLAG-SUMO1 treated with siSUMO1 and doxycycline for 48 h before denaturing lysis and immunoprecipitation with the indicated Mabs. Immunoprecipitated material was divided in two and probed with anti-FLAG or anti-RANGAP1 antibodies. The anti-FLAG panel is from a 30-s exposure, while the anti-RANGAP1 blot was exposed for 1 min. The input for RANGAP1 detects both the unmodified and SUMO1 conjugated species (~ 70 and 100 kDa, respectively). (**b)** Quantitation of FLAG-SUMO1 IP signal (from 70 kDa to the well front) from 6 experiments. The enrichment is shown as a mean % relative to the M2 anti-FLAG IP from the same cell lysate as an internal control for each experiment. Error bars show S.E.M. (**c)** Quantitation of the mean RANGAP1 IP signal (100 kDa species) by antibodies raised to SUMO1 relative to RANGAP1 precipitation by anti-FLAG, M2. Error bars show S.E.M. N = 6. (**d)** Immunoprecipitation of FLAG-SUMO2 by anti-FLAG and MAbs raised to SUMO2/3 from U2OS cells with the ability to express FLAG-SUMO2 (treated as for **a**). (**e)** Quantification of the mean FLAG-SUMO2 IP signal by antibodies raised to SUMO2/3 as a % of those precipitated by anti-FLAG M2, from 5 experiments. Error bars show S.E.M. (**f)** Immunoprecipitation of FLAG-SUMO2 by M2 and SUMO4 MAbs (treated as for **a** but using SUMO4 MAbs). (**g)** Immunoprecipitates by anti-FLAG and anti-SUMO2/3 MAbs, from (**d**), probed for KAP1. The approximate location of mono, di and tri-SUMO modified KAP1 is shown. (**h)** Quantification of the mean relative KAP1 enrichment by anti-SUMO2/3 MAbs relative to anti-FLAG M2, from at least 3 experiments. Error bars show S.E.M. (**i)** Relative IP enrichment for FLAG-SUMO1 (total SUMOylation) versus RANGAP1-SUMO1 enrichment (from data in **b,c**). The IP efficiency of FLAG M2 is set at 100% for both conditions. (**j)** Relative IP enrichment of FLAG-SUMO2 and KAP1 by SUMO2/3 antibodies (from data in **e,f**) relative to anti-FLAG M2.

In the investigation of IP by anti-SUMO1 antibodies, we noted clones BD8B16 and D11 immunoprecipitated FLAG-SUMO1 to levels equivalent to anti-FLAG M2. Two of the anti-SUMO1 MAbs that performed poorly in immunoblot analysis (5B12 and 852620) nevertheless immunoprecipitated FLAG-SUMO1 (Fig. 6a,b). We assessed the ability to enrich SUMO1-modified RANGAP1 and found C9H1, D11, EP298 and Y299 precipitated SUMO1-RANGAP1 as well as, or to a greater degree, than anti-FLAG M2, while 21C7, 4D12, BD8B16, 5B12 and 852620 MAbs precipitated less (Fig. 6a,c).

In the investigation of IP by antibodies raised to SUMO2/3, all but clones 18H8 and SM23/496 performed similarly to M2 for SUMO conjugate immunoprecipitation (Fig. 6d,e). All anti-SUMO4 MAbs immunoprecipitated FLAG-SUMO2 conjugates and, to a lesser degree, free FLAG-SUMO2/3 (Fig. 6f). We next tested the ability of each anti-SUMO2/3 MAb to enrich for KAP1/TRIM28. This protein has high basal auto-SUMOylation activity and is SUMOylated as a monomer, multi-monomer, and polymer^25,27,44,45^ (Fig. 6g,h). Clones 12F3 and 8A2 precipitated more SUMOylated KAP1 than FLAG M2. Mono-SUMOylated KAP1 was only detected by FLAG, 12F3, 1E7 and 8A2 antibodies (Fig. 6g). Several SUMO2/3 MAbs failed to enrich SUMO2ylated KAP1 (18H8, 3H12 and SM23/496). When comparing IP efficiency for total FLAG-SUMO1 with SUMO1ylated RANGAP1 we noted that enrichment of RANGAP1 was not always proportional to overall FLAG-SUMO1 IP efficiency (Fig. 6i), while SUMO2/3 MAbs that were efficient at KAP1 enrichment tended to also perform well in FLAG-SUMO2 immunoprecipitation (Fig. 6j).

## Discussion

**Figure 7:**
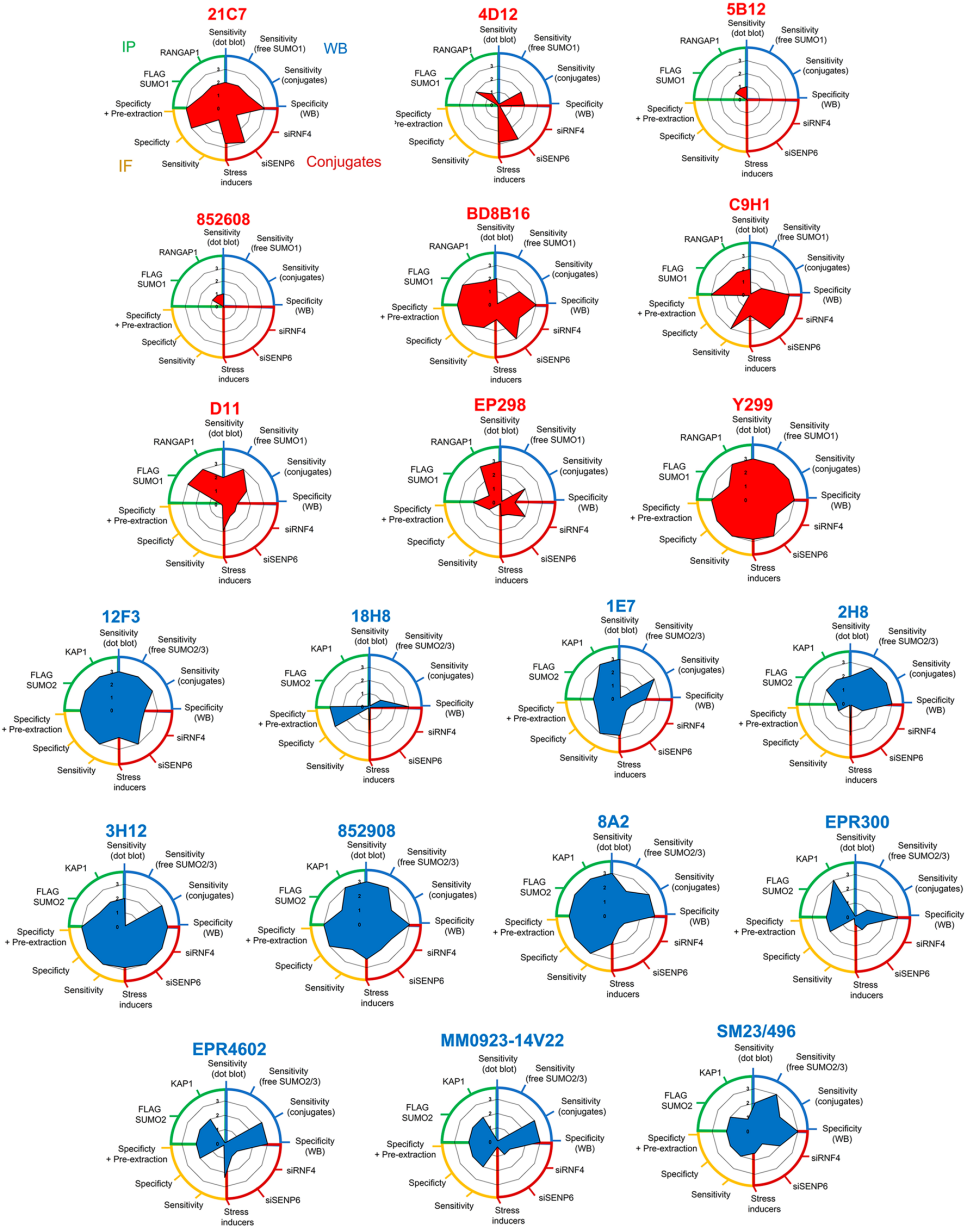
Summary of SUMO MAb performance across multiple applications. Radar plots of SUMO1 (Red) and SUMO2/3 (Blue) MAbs summarising the relative performance for each application tested. Performance is rated from 1 to 3 (low, medium or high), to criteria detailed in the relative quantification section of the materials and methods. Each application is grouped as follows in a clockwise orientation. Blue; performance by immunoblot; dot blot sensitivity, sensitivity (free SUMO), sensitivity (conjugates) and specificity (reduction in conjugates on siSUMO treatment). Red; change in conjugate intensity on siRNF4/siSENP6 treatment, change in conjugates (relative to untreated) for stress inducers summarised as a mean across all tested stresses to give an indication of the dynamics of SUMO conjugates detected following stress. Yellow; indirect immunofluorescence performance on fixed cells; sensitivity (signal intensity), specificity (reduction in the signal by siRNA treatment in both −/+ pre-extraction conditions). Green; immunoprecipitation performance against FLAG-SUMO and RANGAP-1 (SUMO1 MAbs) or KAP1 (SUMO2/3 MAbs).

This study has explored the specificity (reduction in signal on siRNA depletion and cross-reaction between SUMO2/3 and SUMO4) and sensitivity of twenty-four MAbs raised to SUMO family members. We have tested the antibodies against monomeric and conjugated SUMO, in dot-blot, immunoblot, immunofluorescence and immunoprecipitation formats. We describe a tremendous variability between antibodies to detect SUMO states in these applications, with very few 'all-rounders' (Fig. 7). Several companies have committed to improved antibody validation. Abcam, Cytoskeleton and BIORAD have validated their SUMO1 MAbs using *SUMO1* knock-out HAP1 cells. Our cataloguing of nine antibodies raised to SUMO1 finds that several are specific and sensitive in at least one detection format tested. Anti-SUMO1 MAb 852620 (R&D A-716) was not specific by immunoblot (Fig. 2a) but was able to detect SUMO1 in dot blot (Fig. 1b) and immunoprecipitation (Fig. 6a,b). SUMO1 5B12 (MBL Life science) failed in most applications except immunoprecipitation. Two SUMO1 MAbs (EP298 Abcam and D11 Santa Cruz) detect multiple nonspecific bands by immunoblot (Fig. 2a) but, were able to precipitate SUMO1 or RANGAP1 IP (Fig. 6a,b).

Our data suggest that many antibodies raised to SUMO2/3 are sensitive and specific in the formats we tested, although different antibodies are preferable for certain modalities (Fig. 7). SUMO2/3 18H8 (Cell Signalling Technologies) performed poorly in multiple formats, only detecting higher concentrations of recombinant SUMO2 in dot blot (Fig. 1c) and weakly detecting SUMO2 monomers, dimers and polymers in western blots (Fig. 3c). With the exception of Fig. 4b 18H8 also poorly detected SUMO2/3 conjugates by western blots in all other tests. Many SUMO2/3 MAbs detect SUMO4 when overexpressed (Supplemental Fig. 2d–h) but show a preference for SUMO2/3 over SUMO4. We found that the ability to detect high molecular weight SUMO2 polymers did not necessarily correlate with the ability to detect changes in high molecular weight SUMO2/3 following RNF4 or SENP6 depletion (e.g., 1E7, MM0-923 and SM23/496), presumably reflecting the greater complexity of SUMOylation in cells. Like ubiquitin, SUMO1-3 form polymers on multiple internal lysines with evidence of branching and mixed chains composed of other Ubls^26^. An analysis of ubiquitin antibodies highlights the variability of these reagents to detect different homotypic linkages^46^. The same may be true for SUMO MAbs. We also highlight the variation (0–15% of total SUMO) in the detection of monomeric/unconjugated SUMO by the tested MAbs (Fig. 3a) and the inability of some clones (4D12 and EPR300) to detect monomeric SUMO by dot blot (Fig. 1b,c). Where detectable, free SUMO1 and SUMO2/3 levels were reduced by strong conjugate-inducing agents, such as MG132 and heat shock but barely altered by other agents (Fig. 4), suggesting the free pool of SUMO may be depleted under some specific conditions.

All tested antibodies raised to SUMO4 failed to show specificity towards SUMO4 over the closely related SUMO2/3. In some formats, they showed greater selectivity for SUMO2/3 over SUMO4. Unlike other family members, SUMO4 is both nuclear and cytoplasmic (Fig. 5a) and shows no increase in conjugation following cellular stresses (Fig. 4d). Clone EPR7163 (raised against SUMO4) and EPR300 (raised against SUMO2) were rebranded as SUMO2/3/4 antibodies after in-house validation by Abcam. Our data suggest that reports of specific SUMO4 biology using antibodies raised to SUMO4 should be treated cautiously. We tested four of nine SUMO4 MAbs on the market. Those we did not test were antibodies not validated by their vendor (2E4, 9H10-e) or those for which vendors reported nuclear staining patterns inconsistent with SUMO4 (3C3) or were classified as pan SUMO2/3/4 (C3). Collectively, we have generated an extensive validation resource for the SUMO research community. We believe this will guide researchers towards the more robust research reagents available, saving time and valuable resources. We also highlight the inherent variability in SUMO conjugate detection by different SUMO MAbs, likely reflecting the complexity in SUMO signalling and potentially explaining issues with reproducibility between laboratories.

## Materials and methods

### Recombinant SUMO proteins (rSUMO)

Human SUMO1, SUMO2, and SUMO3 cDNA were cloned into pGEX-4T1 using BamHI and EcoR1, or BamH1 and SalI for SUMO1 restriction sites. SUMO4 was cloned into pGEX-6P1 using BamHI and EcoR1. Plasmids were transformed into BL21 (DE3; New England Biolabs) and clones were picked for growth in 40 mL LB (Melford) starter cultures supplemented with ampicillin (100 µg/ml) for overnight growth at 37 °C. For each litre of culture 10 mL of starter culture was added and grown at 37 °C/180 rpm to OD_595_ of 0.6. The temperature was reduced to 18 °C and 0.5 mM IPTG added for a 16-h induction. Bacteria were pelleted at 5000*g* (4 °C) for 10 min and resuspended in lysis buffer (20 mM Tris–HCl pH 8, 130 mM NaCl, 1 mM EDTA, 1 mM EGTA, 1.5 mM MgCl_2_, 1% Triton-X100, 10% Glycerol, 1 mM DTT, Roche complete protease inhibitor) with 0.5 mg/mL Lysozyme and placed on a roller for 45 min at 4 °C. Lysates were sonicated five times for 30 s followed by centrifugation (48,000*g* at 4 °C for 30 min) and passing supernatant through a 0.45 μm syringe filter. Lysates were incubated with 250 μL of GST Sepharose 4B (GE Healthcare) for 3 h at 4 °C. Beads were pelleted and washed three times in 10 mL lysis buffer and once in 10 mL cleavage buffer. For SUMO1-3 thrombin (16 U; Promega) was used for cleavage in buffer (20 mM Tris–HCl pH 8.4, 150 mM NaCl, 1.5 mM CaCl_2_), while PreScission Protease (30 U; SIGMA, in cleavage buffer 50 mM Tris–HCl pH 7, 150 mM NaCl, 1 mM EDTA, 1 mM DTT) was used for SUMO4 owing to a partial thrombin cleavage site unique to SUMO4. 500 μL buffer was added to GST-SUMO1-4 beads with respective cleavage protease and incubated for 16 h at 4 °C with agitation. GST beads were pelleted with centrifugation at 1000*g* at 4 °C for 3 min and the supernatant collected for further centrifugation at 14,000*g* (4 °C) for 20 min. Samples were passed through a Superdex Increase 10/300 GL column (Cytivia) attached to an AKTA pure (UNICORN software) for size-exclusion chromatography equilibrated in 20 mM Hepes pH7.5, 100 mM NaCl, 0.5 mM TCEP. SUMO protein fractions were pooled and aliquoted for storage at −80 °C. SUMO2 dimers (ULC-200-0505) and 2–8 polymers (ULC-210-025) were purchased from R&D.

### Immunoblot

Unless otherwise stated, all cell lysates were made by direct lysis in 6× Laemmli buffer (Alfa Aesar) to inactive SENPs and denature proteins followed by heating at 95 °C for 5 min, sonication and centrifugation. Subcellular fractions were generated according to^47^. Lysates were separated on 4–20% Tris–Glycine Novex wedgewell gels (Invitrogen) followed by wet transfer (stacking and resolving gel) in transfer buffer supplemented with 20% methanol on 0.45 μm PVDF membrane (IMMOBILON-P, Millipore). PVDF was selected over nitrocellulose as it shows an improved transfer of free SUMO^48^. PVDF membranes were stained with Ponceau S stain (Merck) to confirm complete transfer, followed by blocking for 30 min at room temperature in 5% non-fat milk in PBST (0.01% Triton-X100). Membranes were probed with primary antibody (1 µg/mL or 1:1000) with gentle rolling at 4 °C for 16 h followed by 3 × 10 min washes with PBST and incubation with secondary HRP antibodies (1:10,000) in 5% milk/PBST for 2 h at room temperature. Following 3× 10-min PBST washes, membranes were exposed to ECL reagent (EZ-ECL, GeneFlow) for two minutes prior to exposure with blue X-ray film (Wolflabs). Details of antibodies used are in supplemental Table 3. Uncropped blots with regions shown in the figure for each exposure are included in the supplemental data. Densitometry was performed using Image J on multiple film exposures to ensure linearity of signal.

### Dot blots

rSUMO proteins were diluted to 3 µg/mL in 6× Laemmli buffer and diluted serially 1:2 10 times to a final dilution of 3 ng/mL. After methanol activation, PVDF membranes were dried and dotted with 1 µL of denatured rSUMO. After drying at room temperature for 30 min, blots were probed as for immunoblots. Each blot was exposed to X-ray film at four exposure time points (0.5, 1, 2 and 10 min).

### Peptide competition

For peptide competition assays 12F3, 2H8, 852908, 8A2, 21C7, ARC1382, EPR7163, JJ-085, IOO-19, 5B12, BD8B16, C9H1, D11, Y299 and MM0-923 were measured by peptide competition dot blot as previously described^25^. As some MAbs performed poorly by dot blot (EPR4602, EPR300) the same protocol was adapted to immunoblot competitions with U2OS lysates separated on 6% SDS-PAGE gels using the same concentration of peptide as for dot blots. The peptide sequences can be found in supplemental Table 4, all peptides were synthesized by GenScript.

### Cell culture

6× His-FLAG SUMO1, SUMO2 and FLAG-HA SUMO4 FlpIn stable cell lines were generated using U2OS^TrEx-FlpIn^ (a gift from Grant Stewart, University of Birmingham) cells transfected with pcDNA5/FRT/TO based vectors (Supplemental Table 2) and the recombinase pOG44 (Invitrogen) using FuGene6 (Promega) at a ratio of 4 µl FuGENE/µg DNA. After 48 h, cells were selected into hygromycin-supplemented media (100 µg/ml) and grown until colonies formed on plasmid-transfected plates but not controls. Knockdowns by siRNA were performed as for^36^ (sequences in supplemental Table 4). Details of the cell lines, including culture conditions and their sources, can be found in supplemental Table 5. Details of the drugs used can be found in supplemental Table 6.

### Immunofluorescence

Cells were plated at 2.5 × 10^4^ cells/well on 13 mm glass coverslips in 24 well plates (Corning) and attached overnight prior to siRNA depletion for 48 h. For pre-extraction after 1× PBS wash cells were treated with 250 µL/well ice cold CSK buffer (100 mM NaCl, 300 mM sucrose, 3 mM MgCl_2_, 0.7% Triton-X100 and 10 mM PIPES) for 30 s prior to fixation with 4% Paraformaldehyde (PFA) in PBS at room temperature for 10 min. For non-pre-extracted samples, cells were fixed in 4% PFA at room temperature for 10 min followed by permeabilization with 0.5% Triton-X100 in PBS for 5 min. Coverslips were blocked with 5% FBS in PBS for 1 h at room temperature, followed by incubation with primary antibodies at 1 µg/mL (or 1:1000 for recombinant and CST MAbs) overnight at 4 °C in 5% FBS. Coverslips were washed twice with PBS followed by incubation with Alexa-Fluor 555 conjugated secondary antibodies at 1:2500 for 2 h at room temperature in the dark. Cells were washed twice with PBS prior to incubation with 250 µL of Hoechst (1 µg/mL) for 2 min. Coverslips were mounted on slides using Immuno-Mount (Thermo Scientific) and sealed. Imaging was carried out on a Leica DM6000B microscope using an HBO lamp with 100 W mercury short arc UV bulb light source. Images were captured at each wavelength sequentially using the Plan Apochromat HCX 100×/1.4 Oil objective at a resolution of 1392 × 1040 pixels. Exposure conditions were kept consistent within each panel of SUMO MAbs.

### Denaturing SUMO immunoprecipitations

U2OS FlpIn 6xHIS-FLAG SUMO1 or SUMO2 cells were plated on 15 cm^2^ plates at 5 × 10^6^ for 24 h prior to siRNA depletion with either 10 nM of each SUMO1 siRNA or 10 nM each of SUMO2 and SUMO3 siRNA (SIGMA). Doxycycline (1 µg/mL) was added concurrently with siRNA depletion for 48 h. Cells were trypsinized and pelleted in cold PBS followed by lysis in 1% SDS Lysis buffer (20 mM Sodium phosphate pH 7.4, 150 mM NaCl, 1% SDS, 1% Triton-X100, 0.5% Sodium deoxycholate, 5 mM EDTA, 200 mM IAA and complete Protease and Phosphatase inhibitors). Lysates were sonicated 3× for 20 s, followed by the addition of 50 mM DTT and boiling for 10 min. Lysates were clarified by centrifugation and frozen at −80 °C for later use. For immunoprecipitation, Protein A/G Plus agarose beads (Pierce) were washed twice in 10× packed bead volumes of RIPA buffer (20 mM Sodium phosphate pH 7.4, 150 mM NaCl, 1% Triton-X100, 0.5% Sodium deoxycholate, 5 mM EDTA, 100 mM NEM and Protease/Phosphatase inhibitor cocktail). Antibodies were bound at 4 µg/30 µL of beads at 4 °C for 3 h. 1% SDS lysates were thawed and diluted 10× with RIPA buffer before incubating 1 mL of lysate with MAb conjugated beads overnight at 4 °C. A fraction of lysate was retained as input, and the beads were washed 3× with 1 mL of RIPA buffer before eluting in 30 µL of 6X Laemmli buffer and boiling.

### Statistics

Unless otherwise stated all statistical analysis was by two-sided Students T-test throughout. *p < 0.05, **p < 0.01, ***p < 0.005 ****p < 0.001. All centre values are given as the mean and all error bars are standard error about the mean (s.e.m). Data was analyzed using GraphPad Prism 7.03.

### Quantification

All immunoblot or Image analysis for quantification was done using ImageJ unless otherwise specified.

### Relative quantification (Fig. 7)

*Sensitivity by dot blot,* MAbs that produced a saturating signal (less than 25% change between two exposure time points) across at least the five most concentrated dots at the 10-min exposure were classed as having high sensitivity by dot blot (score = 3). Those that produced a saturating signal on at least two concentrations at 10 min of exposure were classed as moderate (score = 2). MAbs that did not reach saturation at any concentration or exposure time were classed as low (score = 1). MAbs that did not produce any signal were scored as 0.

*Sensitivity (free SUMO)*, sensitivity for the detection of free SUMO by immunoblot was scored as 3 if MAbs detected > 10% of free SUMO, 2 is they detected 5–10% and 1 if they detected 0–5%. MAbs that did not detect any free SUMO were scored as 0.

*Sensitivity (conjugates),* sensitivity for the detection of SUMO conjugates by immunoblot was scored as 3 if the signal intensity (by densitometry of conjugates migrating > 70 kDa) showed no change (saturation) between two exposure time points. Scores of 2 if a change of less than 50% between two exposures and 1 if conjugate signal failed to saturate at the 10 min timepoint.

*Specificity (WB)* was classed as high (score = 3) if the signal from either the siNTC or complemented lanes were at least 4× higher than the siSUMO lane. Moderate specificity (score = 2) was classed as any non-specific bands detected by the MAb on siSUMO treatment. Low specificity was for MAbs that showed no difference between control and siSUMO treatment (score = 1).

*Changes in SUMO conjugates on RNF4 or SENP6 siRNA depletion* were assessed as high (3) if either RNF4 or SENP6 depletion caused a > 100% increase in conjugate levels versus FLAG-SUMO, moderate (2) for > 50% increase or low (1) for less than 10%.

*Stress inducers*; conditions that caused a % increase in SUMO conjugates (relative to untreated cells and excluding free SUMO) of less than 10% were scored as 0, 11–50% scored as 1, 51–100% scored as 2 and greater than 100% as a 3. The mean across thirteen stress inducers is shown.

*Sensitivity by immunofluorescence* was measured on the mean fluorescence intensity in siNTC cells (Arbitrary units). Less than 2000 was classed as low sensitivity (1), between 2000 and 4000 was classed as moderate sensitivity (2) and higher than 4000 was classed as high sensitivity (3).

*Specificity by immunofluorescence (−/ + pre-extraction)* was classed as high (3) if the signal between siNTC and siSUMO was > 3×, moderate (2) if > 2× and low (1) if the signal was not significantly reduced under either condition.

*IP efficiency* (FLAG SUMO1 or FLAG SUMO2) was measured relative to the FLAG-SUMO control. High efficiency (3) was classed if 100% or more of the IP signal was detected, moderate (2) if 50% and low (1) if less than 25%.

*RANGAP1 and KAP1 IP efficiency* was measured as for total SUMO IP efficiency.

## Supplementary Information

Supplementary Information.

## Data Availability

The datasets used and/or analyzed during the current study are available from the corresponding author on reasonable request.
