# Supplementary material for: SUMO monoclonal antibodies vary in sensitivity, specificity, and ability to detect types of SUMO conjugate
Source: Sci Rep. 2022 Dec 9;12:21343. doi: 10.1038/s41598-022-25665-6 (PMC9734647; doi:10.1038/s41598-022-25665-6)

Supplemental data, methods and tables

SUMO monoclonal antibodies vary in sensitivity, specificity, and ability to detect types of SUMO conjugate.

Alexander J. Garvin[1*], Alexander J. Lanz[1], Joanna R. Morris[1*]

1. Birmingham Centre for Genome Biology and Institute of Cancer and Genomic Sciences, College of Medical and Dental Schools, University of Birmingham, B15 2TT, UK.

* Correspondence; a.j.garvin@bham.ac.uk, j.morris.3@bham.ac.uk

Supplemental Figure 1

a

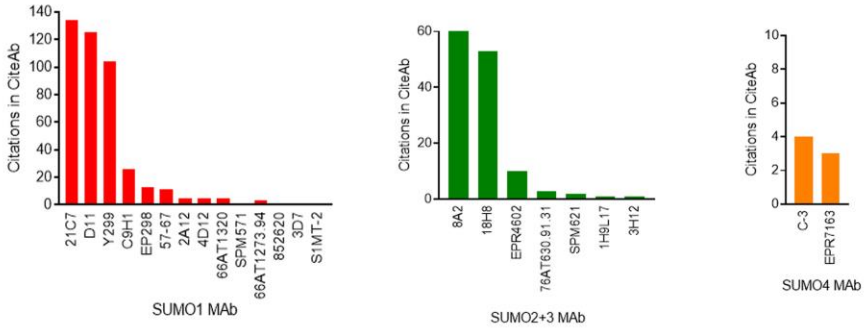

b

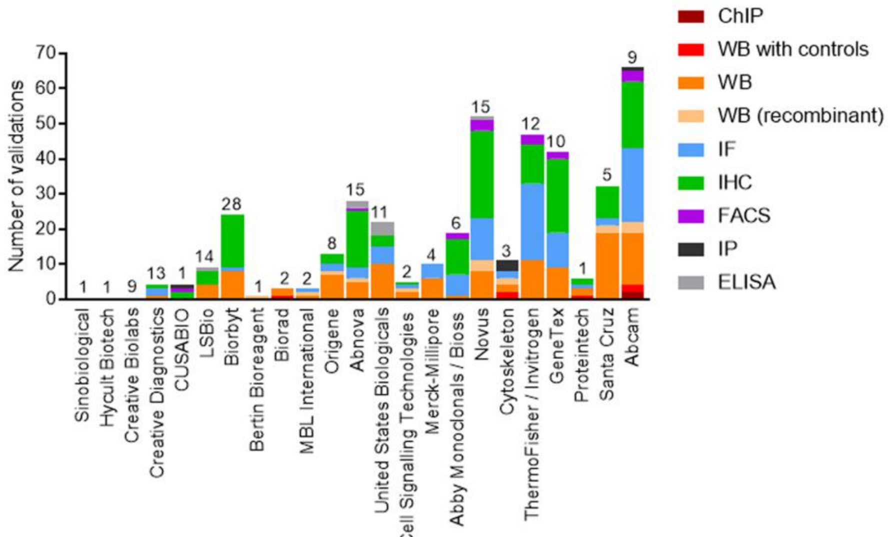

c

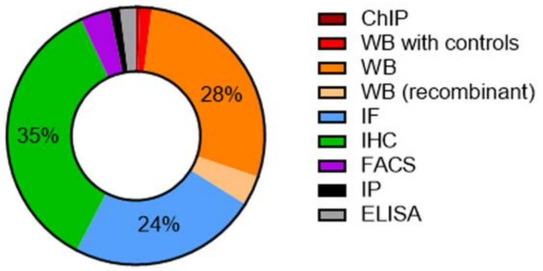

Supplemental Figure 1. SUMO MAbs vary in validation status.

a. Citations for each SUMO MAb from the CiteAb database as of March 2022.

b. Total number of validations for SUMO1, SUMO2/3 and SUMO4 MAbs ranked by the vendor. The total number of SUMO MAbs available per vendor (as of March 2022) is shown above each bar. Validations include any supporting images or data available on the products webpage including those generated by the vendor. Reviews on the product webpage were only included if they contained accompanying images or data. Types of validation are colour coded.

c. Type of validation across all SUMO MAbs and vendors. Controls include either negative (knockdown or knockout) or positive (over-expression or induced expression with stress). Abbreviations, ChIP (Chromatin Immunoprecipitation), WB (Western blot), recombinant – testing on *E. coli* derived recombinant SUMO proteins, IF (Immunofluorescence), IHC (Immunohistochemistry), FACS (Fluorescence Activated Cell Sorting), IP (Immunoprecipitation), ELISA (enzyme-linked immunosorbent assay).

Supplemental Figure 2

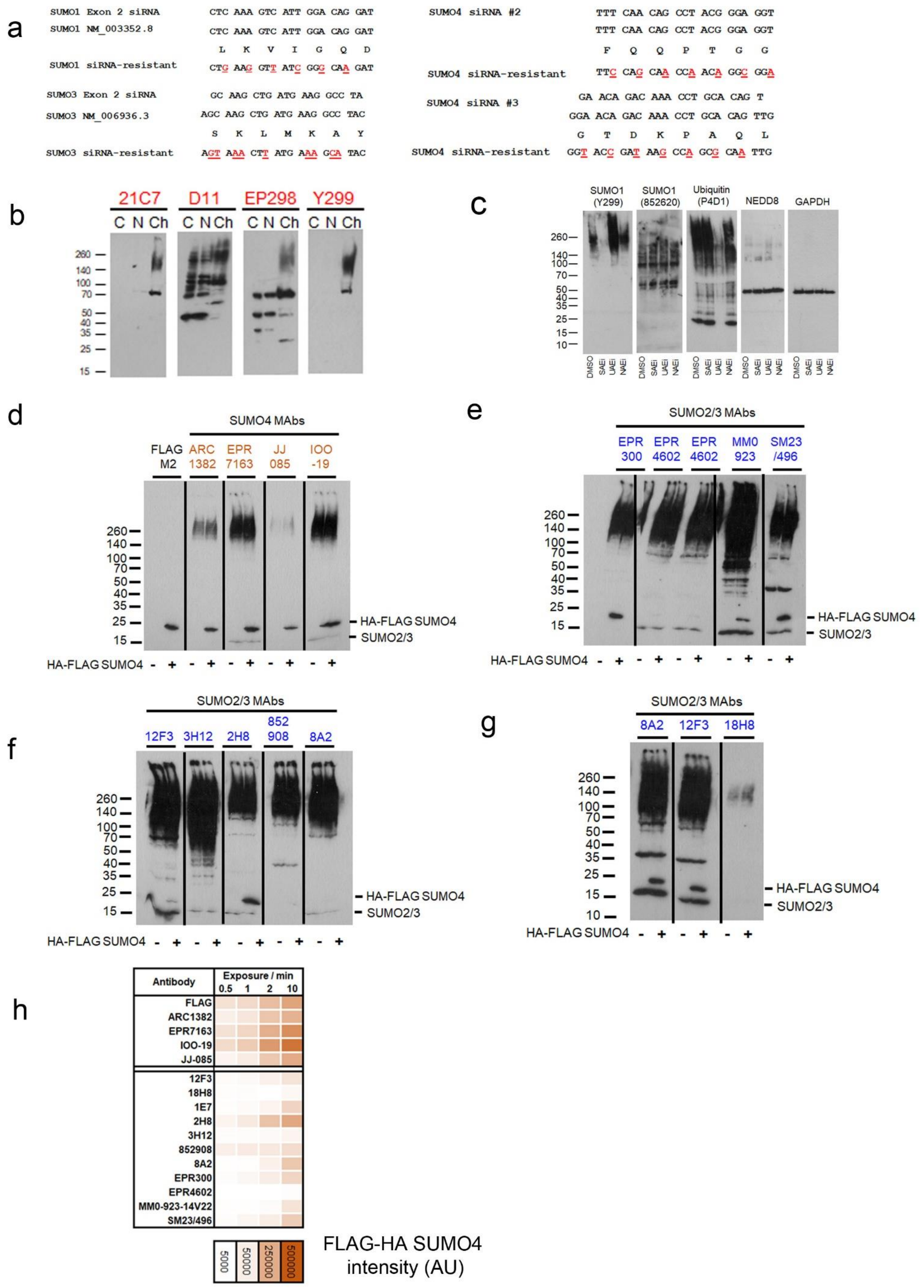

Supplemental Figure 2. Non-specific and cross-reactive features of SUMO MAbs.

a. Location of synonymous mutations in SUMO1, SUMO3 and SUMO4 cDNA to generate siRNA resistant species.

b. U2OS cell lysates separated by fractionation into cytoplasm (C), soluble nuclear (N) or chromatin (Ch) were probed with 21C7 and Y299 control SUMO1 MAbs and two SUMO1 MAbs that detect non- specific bands (D11 and EP298).

c. U2OS treated for six hours with 10 µM each of ML-792 (SUMO E1 inhibitor), TAK-243 (Ubiquitin E1 inhibitor) or MLN4924 (NEDD8 E1 inhibitor) prior to lysis (400 µL 6X Laemmli buffer / 6 well), separation on 4-20% SDS PAGE gels (20 µL / lane) and probed the antibodies shown.

d, e, and f. U2OS cells expressing FLAG-HA SUMO4 untreated (-) or doxycycline treated (+) for 48 hours prior to lysis in Laemmli buffer. PVDF membranes divided (indicated by black lines) and probed with indicated MAbs but imaged side by side for comparison. Two-minute film exposures shown.

g. As for 2d-f but 10-minute exposure shown. 8A2 and 12F3 probed separately but exposed to film with the 18H8 panel are shown for comparison.

h. Heat map showing the quantified signal of over-expressed FLAG-HA SUMO4 (band ~ 20 kDa) in U2OS lysates by antibodies raised against SUMO2/3 and 4. Detection by FLAG M2 as an internal control. Intensity is shown across all four film exposure times with deeper orange signifying higher FLAG-HA SUMO4 detection. High molecular weight conjugates and endogenous SUMO2/3/4 (band at ~15 kDa) were not included in the quantification.

Supplemental Figure 3A

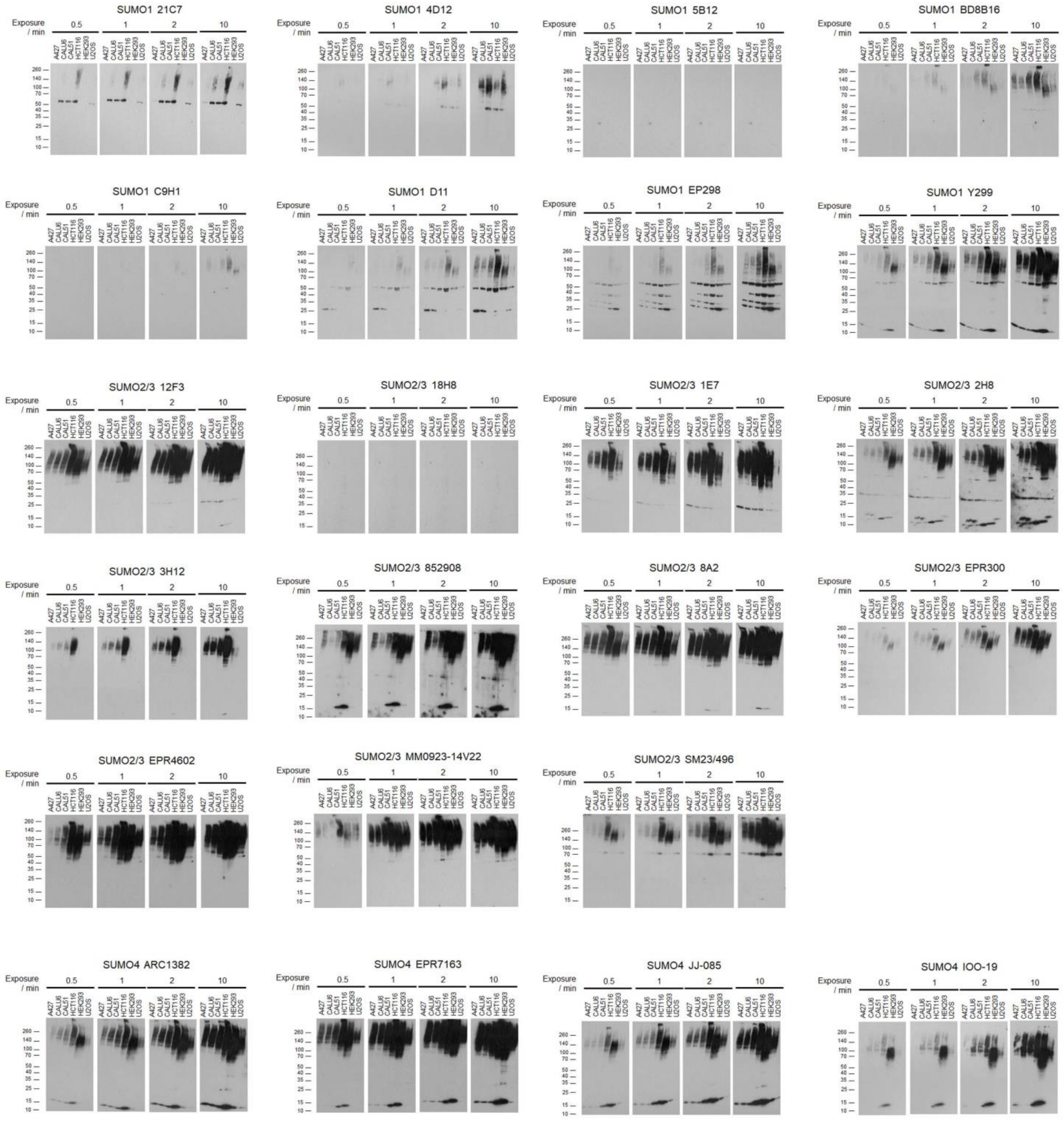

Supplemental Figure 3a. All exposures of immunoblots related to Figure 3a and 3b

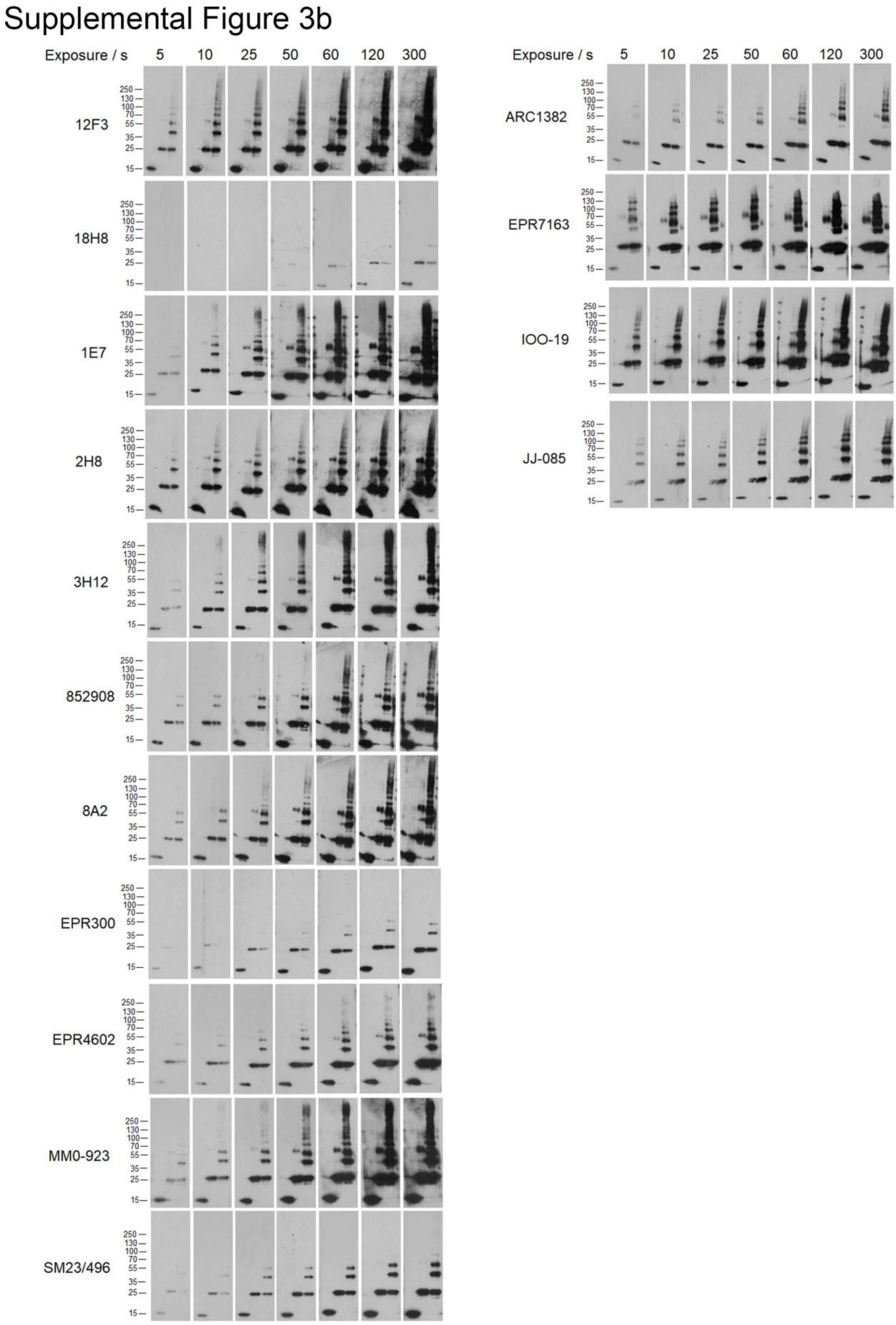

Supplemental Figure 3b. All exposures of immunoblots related to Figure 3c

Supplemental Figure 4a

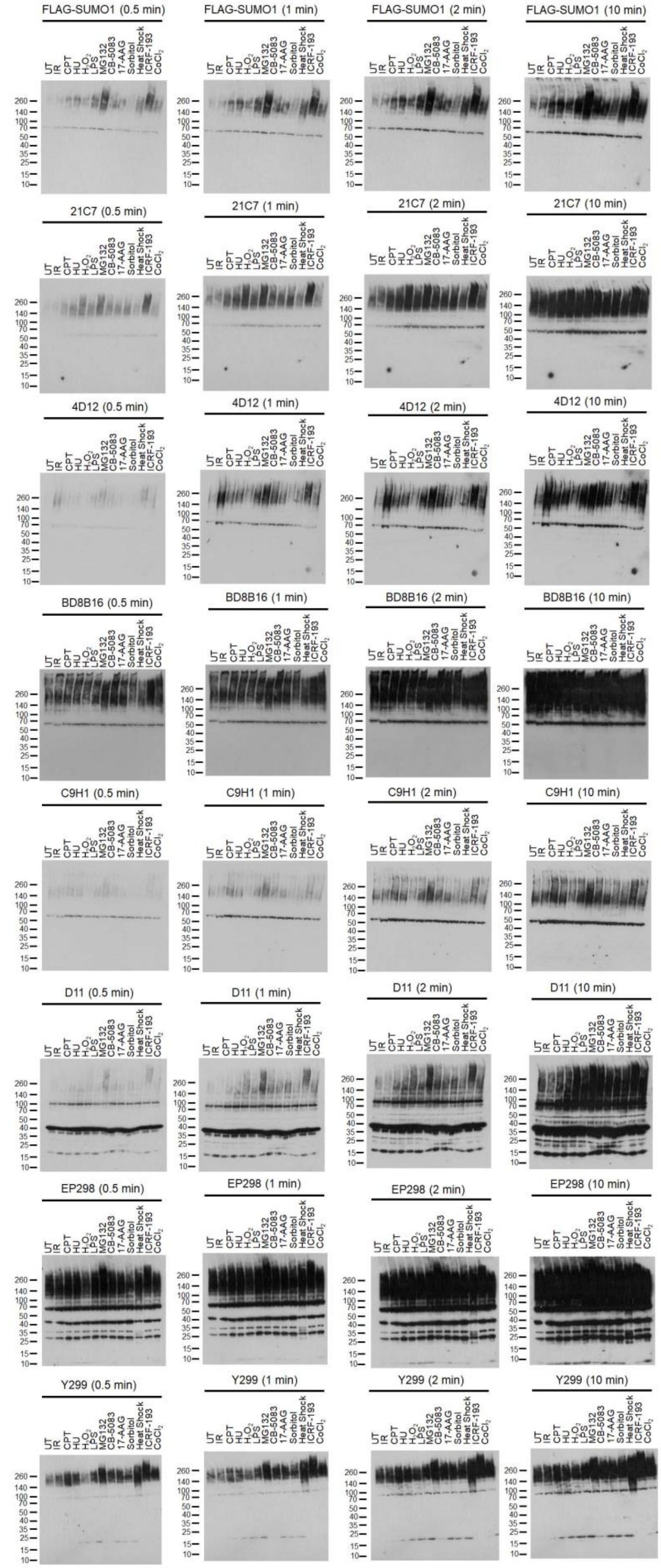

Supplemental Figure 4a. All exposures of SUMO1 immunoblots related to figure 4b.

Supplemental Figure 4b

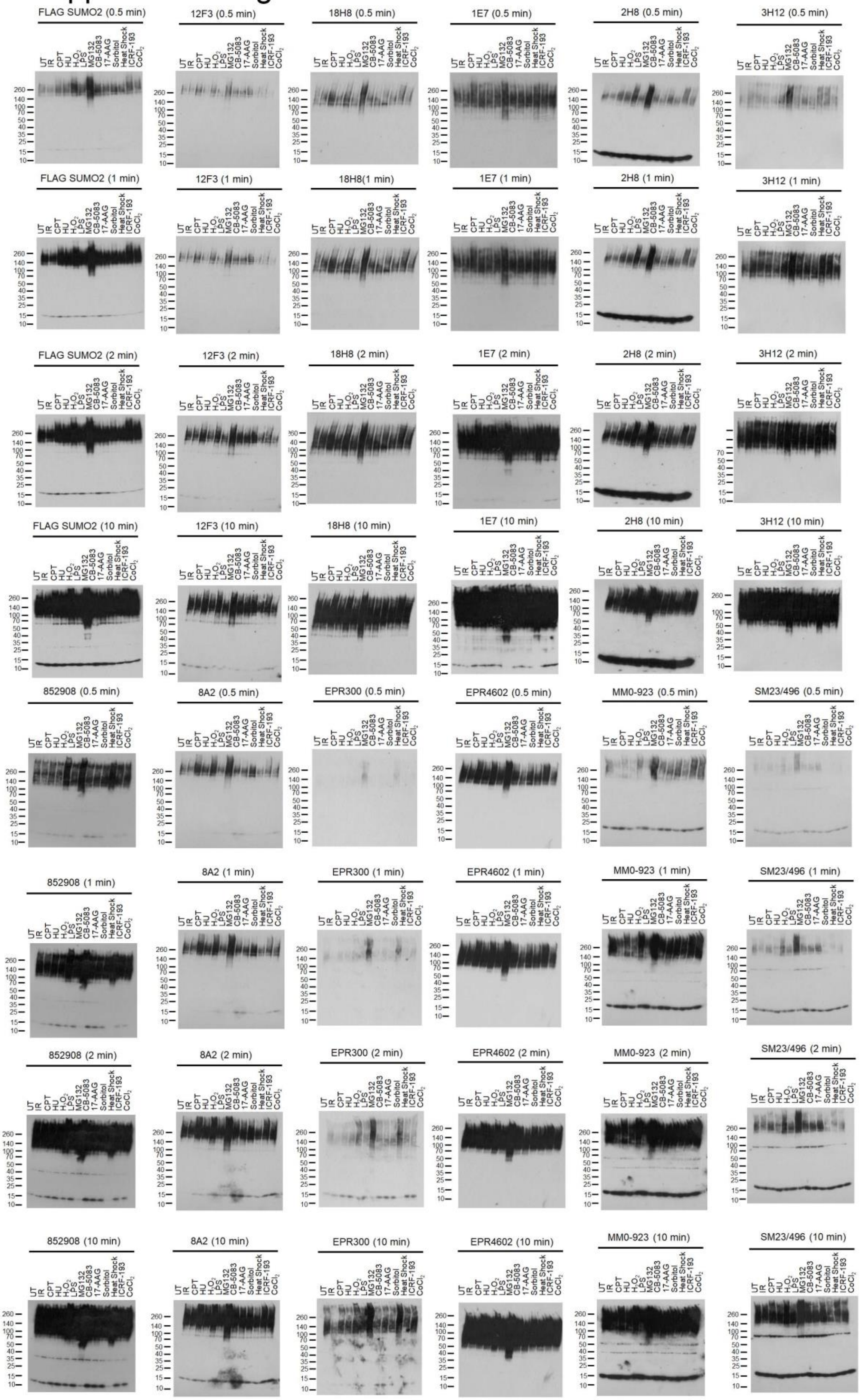

Supplemental Figure 4b. All exposures of SUMO2/3 immunoblot related to Figure 4b.

Supplemental Figure 4c

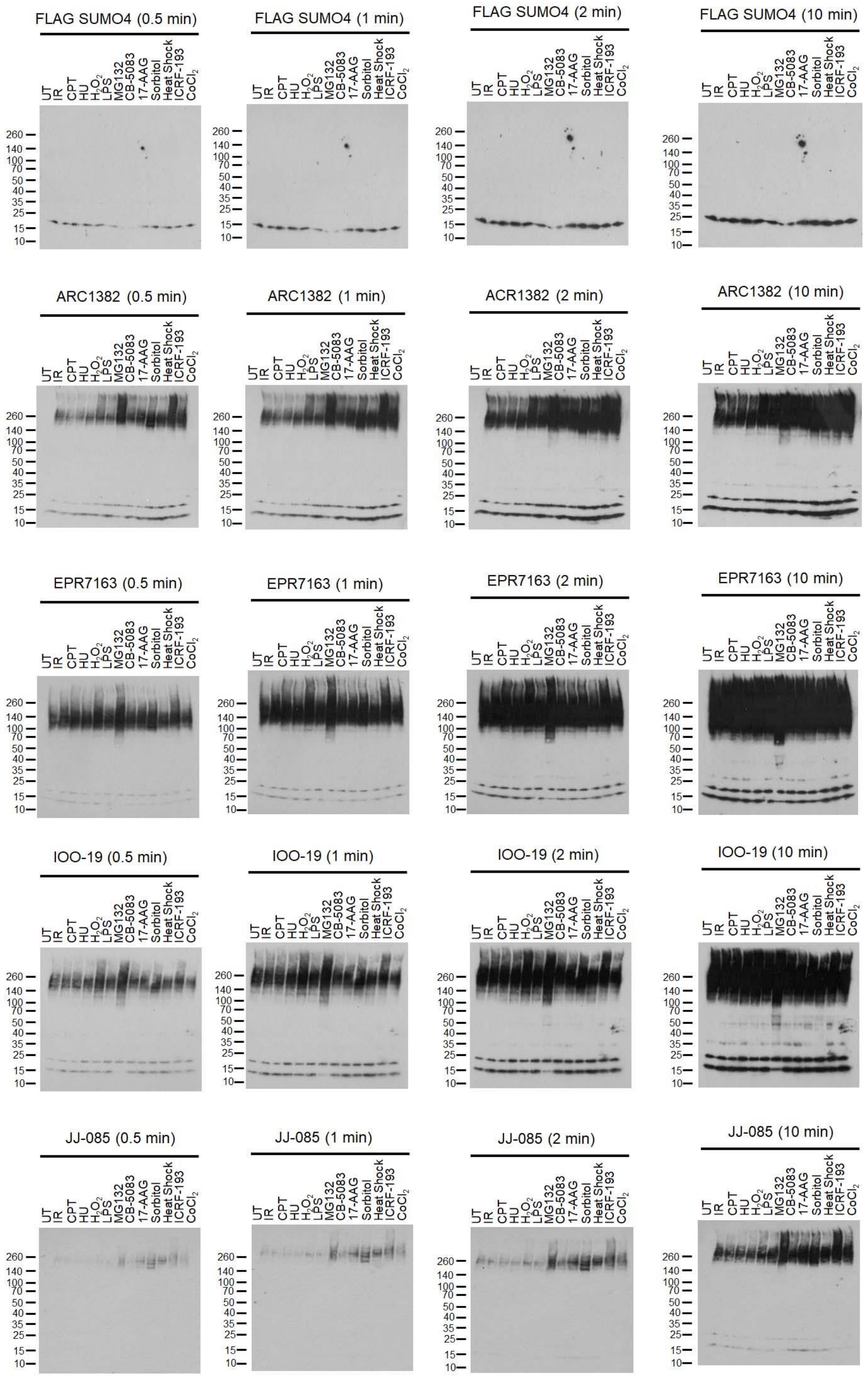

Supplemental Figure 4c. All exposures of SUMO4 immunoblot related to Figure 4d.

Supplemental Figure 5

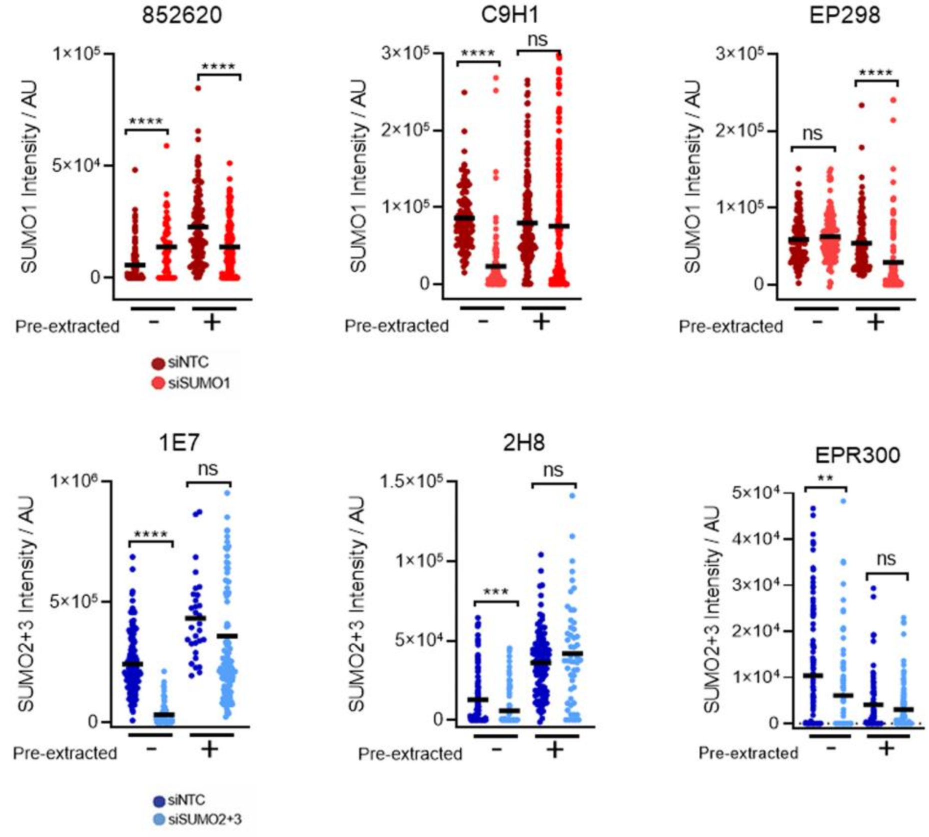

Supplemental Figure 5. Fixation can affect specificity of SUMO MAbs

SUMO immunofluorescent intensity (AU) detected by indicated MAbs in U2OS cells (siNTC or siSUMO treated) fixed with PFA prior to Triton X-100 permeabilisation (-) or pre-extracted with CSK buffer prior to fixation (+). Statistical significance by two tailed students t-test is indicated. Ns = not significant. N= ~100 cells per condition.

Supplemental Tables

Supplemental Table 1. Commercially available SUMO1, SUMO2/3 and SUMO4 Monoclonal antibodies

Clone Name	Target	Vendor / cat number	Host	Immunogen[1]	Isotype	RRID
3	SUMO1	Sinobiological (13095-R003)	Rabbit	rSUMO1 Ser2-Val101	IgG	AB_2786592
144	SUMO1	Creative Diagnostics (CABT-37091MH)	Mouse	Not stated		AB_2357980
10i67	SUMO1	US Biologicals (S0750-01 Mab)	Mouse	GST-SUMO1	IgG1	
1B12-E2	SUMO1	Hycult Biotech	Mouse	hSUMO1-Lipoplex O	IgG3	
1C6	SUMO1	Bertin Bioreagent (G01063)	Mouse	Ovalbumin conjugated synthetic peptide	IgG2b κ	
1SUM-1C6	SUMO1	Invitrogen (MA3-088)	Mouse	SUMO1 aa 85-97 peptide	IgG1κ	AB_2633333
19A156	SUMO1	US Biologicals (506063 Mab)	Rabbit	rSUMO1	IgG	
21C7	SUMO1	DSHB	Mouse	His-SUMO1	IgG2b	AB_2198257
2C4A11	SUMO1	Westburg	Rabbit	hSUMO1 peptide		
3D7	SUMO1	SIGMA (SAB1402954)	Mouse	GST-SUMO1	IgG2aκ	AB_10760295
3B5B5	SUMO1	Proteintech (67559-1-Ig)	Mouse	SUMO1 fusion protein Ag29402	IgG1	AB_2882773
4D12	SUMO1	Abcam (ab179907)	Rat	GST SUMO1 1-100 Hs FL	IgG2aκ	AB_10643976
5B12	SUMO1	MBL International (M113-3)	Mouse	rSUMO1 full length		AB_592768
5G3	SUMO1	CUSABIO (CSB-RA022948A0HU)	Rabbit	SUMO1 peptide	IgG	
5D8B16	SUMO1	Cytoskeleton (ASM01)	Mouse	rSUMO1	IgG2b	AB_2884966
6A4	SUMO1	Abby Monoclonals (bsm-51020M)	Mouse	GST-SUMO1	IgG1	
6a183	SUMO1	LSBio	Mouse			AB_798992
66AT1273.94	SUMO1	Biorbyt (orb166941)	Mouse	GST-SUMO1	IgG1	AB_10989506
66AT1273.94.49	SUMO1	BIORAD (VMA00306)	Mouse	GST-SUMO1	IgG1	
66AT1320	SUMO1	Biorbyt (orb166942)	Mouse	GST-SUMO1	IgG1	AB_10980242
76-86	SUMO1	DSHB	Mouse	76-86 (CIADNHTPKLELGMEEEDVIEVYQEQT)	IgG1κ	AB_2618115
7H5	SUMO1	Abby Monoclonals (bsm-52377R)	Mouse	rSUMO1	IgG	
852620	SUMO1	Novus (A716)	Rat	rSUMO1	IgG2a	AB_2490530
A13261	SUMO1	Antibodies Online (A13261)	Rabbit	hSUMO1 peptide	IgG	
ARC0215	SUMO1	ThermoFisher (MA5-35272)	Rabbit	hSUMO1 peptide	IgG	AB_2849174
BFI-19	SUMO1	Abnova (MAB20757)	Rabbit	hSUMO1 peptide	IgG	
C9H1	SUMO1	Cell Signalling Technologies (4940S)	Rabbit	hSUMO1 peptide (N terminal)	IgG	AB_2302825

CBACN-538	SUMO1	Creative Diagnostics (MRO-1452-CN)	Rabbit	rSUMO1	IgG	
D11	SUMO1	Santa Cruz Biotechnologies	Mouse	rSUMO1	IgG3κ	AB_628300
EP298	SUMO1	Abcam (ab133352)	Rabbit	Synthetic peptide	IgG	AB_2240112
FQ309	SUMO1	Creative Diagnostics (DCABH-2692)	Rabbit	Synthetic peptide		
GT1098	SUMO1	GeneTex (GT1098)	Rabbit	Not stated	IgG	
opof	SUMO1	Creative Biolabs (MOB-1981CT)	Mouse	Not stated	IgG3	
OTI2B3	SUMO1	Invitrogen (TA811053)	Mouse	rSUMO1	IgG2b	
OTI1E8	SUMO1	Invitrogen (TA811055)	Mouse	rSUMO1	IgG1	
S1-MT-2	SUMO1	NSJ Bioreagents (V7067)	Mouse	rSUMO1	IgG1κ	
S10G1	SUMO1	Bioss (bsm-51457M)	Mouse	GST-SUMO1	IgG1	
SJ20-03	SUMO1	Novus (NBP2-67419)	Rabbit	rSUMO1	IgG	
SM1/495	SUMO1	Biorbyt (orb388767)	Mouse	rSUMO1	IgG1κ	
SPM571	SUMO1	Biorbyt (orb388768)	Mouse	rSUMO1	IgG1	
SUMO1/1188	SUMO1	Biorbyt (orb388769)	Mouse	rSUMO1	IgG1κ	
T.243.0	SUMO1	Invitrogen (MA5-14877)	Rabbit	hSUMO1 peptide (N terminal)	IgG	AB_10979860
TK31-14	SUMO1	Creative Diagnostics (CABT-L626)	Rabbit	rSUMO1	IgG	
U.354.0	SUMO1	Creative Diagnostics (CABT-L1615)	Rabbit	N terminal peptide	IgG	
Y299	SUMO1	Abcam (ab32058)	Rabbit	C terminal peptide	IgG	
1E7	SUMO2	MBL International (M114-3)	Mouse	rSUMO2	IgG2b	
084DU9.2.2	SUMO2	Creative Diagnostics (DCABY-1339)	Mouse	C terminal peptide	IgG2b	
12F3	SUMO2	Cytoskeleton	Mouse	rSUMO2 +CQIRFRFDGQPINE	IgG	
13H363	SUMO2	US Biologicals	Mouse	rSUMO2	IgG2bκ	
16C92	SUMO2	US Biologicals	Mouse	rSUMO2 1-93	IgG2bκ	
2C7-1A11	SUMO2	Abnova (H00006613-M02)	Mouse	GST-SUMO2	IgG1κ	
2H8	SUMO2	Abnova (H00006613-M06)	Mouse	GST-SUMO2	IgG	
3H12	SUMO2	Abcam (ab191492)	Rat	SUMO2 1-92	IgG	
8A2	SUMO2	DSHB	Mouse	rSUMO2	IgG2b	
973CT8-1-1	SUMO2	Biorbyt (orb134982)	Mouse	His-SUMO2 C terminus	IgG2b	
9B3	SUMO2	Creative Diagnostics (DCABH-10002)	Mouse	rSUMO2	IgG	
ARC1186	SUMO2	Abnova (MAB22897)	Rabbit	Synthetic peptide human SUMO2	IgG	AB_2849531
AT10F1	SUMO2	Abcam (ab104123)	Mouse	SUMO2 1-93	IgG2bκ	
BU21G2	SUMO2	Creative Diagnostics (DCABH-33)	Mouse	rSUMO2 aa 1-93	IgG2b	

EPR300	SUMO2	Abcam (ab109196)	Rabbit	SUMO2 peptide	IgG	
EPR4602	SUMO2	Abcam (ab109005)	Rabbit	SUMO2 peptide	IgG	
KK198-15	SUMO2	Creative Diagnostics (CABT-L819)	Rabbit	rSUMO2		
S23MT-1	SUMO2	Biorbyt (orb56418)	Mouse	rSUMO2	IgG1κ	
SM23/496	SUMO2	Abcam (ab190757)	Mouse	rSUMO2	IgG1κ	
SPM572	SUMO2	Biorbyt (orb388658)	Mouse	rSUMO2	IgG1κ	
SPM621	SUMO2	Biorbyt (orb56184)	Mouse	rSUMO2	IgG1κ	
SUMO2/1199	SUMO2	Biorbyt (orb388659)	Mouse	rSUMO2	IgG1κ	
401504	SUMO3	R & D Systems (A-724)	Rat	rSUMO3 1-103	IgG1	
852908	SUMO3	R & D Systems (A-718)	Rat	rSUMO3	IgG2a	
907614	SUMO3	Adipogen (907614)	Rat	SUMO3 peptide	IgG2a	
18H8	SUMO3	Cell Signalling Technologies (4971S)	Rabbit	N terminal peptide	IgG	
1H9L17	SUMO3	Invitrogen (700186)	Rabbit	SUMO3 peptide 91-103	IgG	
2I0M28	SUMO3	Creative Diagnostics (CABT-L1617)	Rabbit	SUMO3 peptide 91-103	IgG	
4B9	SUMO3	Abnova	Mouse	GST-SUMO3 (partial)	IgG2bκ	
4G11	SUMO3	Sigma Aldrich (SAB1400264)	Mouse	GST-SUMO3 (partial)	IgG2aκ	
76AT630.91.31	SUMO3	Invitrogen (MA5-11148	Mouse	GST-SUMO3	IgG1	
J.778.2	SUMO3	Invitrogen (MA5-14951)	Mouse	SUMO3 peptide	IgG	AB_10979052
K.889.3	SUMO3	Creative Diagnostics (CABT-L1616)	Rabbit	N terminal peptide	IgG	
LW-M2	SUMO3	Santa Cruz (sc-135572)	Mouse	rSUMO3	IgG2aκ	
MM0923-14V22	SUMO3	Abcam (ab201594)	Rat	rSUMO3 1-103	IgG2	
S3G2	SUMO3	Bioss (bsm-51458M)	Mouse	GST-SUMO3	IgG1	
1A1B3	SUMO2/3	Proteintech (67154-1-Ig)	Mouse	SUMO2/3 fusion protein Ag28672	IgG2b	
7G10	SUMO2/3	Bioss (bsm-52716R)	Rabbit	C terminal SUMO2+3	IgG	
IBG-19	SUMO2/3	Abnova (MAB20758)	Rabbit	Peptide corresponding to human SUMO2/SUMO3	IgG	
JJ-087-04	SUMO2/3	ThermoFisher (MA5-32427)	Rabbit	C terminus of SUMO2+3	IgG	AB_2809705
2E4	SUMO4	Creative Diagnostics (DCABH-13669)	Mouse	GST-SUMO4 NP_001002255.1	IgG2a	
3C3	SUMO4	Bioss (bsm-52767R)	Mouse	rSUMO4 1-100	IgG	
9H10-e	SUMO4	Kerafast (EFH003)	Mouse	rSUMO4		
ARC1382	SUMO4	Invitrogen (MA5-35745)	Rabbit	SUMO4 peptide	IgG	AB_2849645

C3	SUMO4	Santa Cruz (sc-393144)	Mouse	rSUMO4 1-95	IgG	
EPR7163	SUMO4	Abcam (ab232032)	Rabbit	SUMO4 peptide	IgG	
IOO-19	SUMO4	Abnova (MAB20759)	Rabbit	SUMO4 peptide	IgG	
JJ-085	SUMO4	Abnova (MA5-32478)	Rabbit	rSUMO4 1-100	IgG	AB_2809755
KK196-12	SUMO4	Creative Diagnostics (CABT-L851)	Rabbit	rSUMO4	IgG	
852721	Pan SUMO	R&D (A-714)	Rat	rSUMO2	IgG2a	

1. Abbreviations

hSUMO; immunogen is derived from a human SUMO protein sequence, but no other information is stated on the datasheet.
rSUMO; recombinant / bacterially derived SUMO proteins, the N terminal tag is not stated.
GST-SUMO; Glutathione-S-transferase N terminally fused SUMO proteins, presumably recombinant.
His-SUMO; Histidine tagged N terminally fused SUMO proteins, presumably recombinant.

Supplemental Table 2. Plasmids

**Plasmid Name**	**Source**
pCDNA5/FRT/TO 6XHis-FLAG SUMO1 WT	This paper / GenScript
pCDNA5/FRT/TO 6XHis-FLAG SUMO2 WT	This paper / GenScript
pCDNA5/FRT/TO 6XHis-FLAG SUMO3 WT	This paper / GenScript
pCDNA5/FRT/TO FLAG-HA SUMO4 WT	This paper / GenScript
pCDNA5/FRT/TO FLAG-HA SUMO4 M55V	This paper / GenScript
pGEX-4T1 ProSUMO1	This paper / GenScript
pGEX-4T1 SUMO1 WT	This paper / GenScript
pGEX-4T1 ProSUMO2	This paper / GenScript
pGEX-4T1 SUMO2 WT	This paper / GenScript
pGEX-4T1 ProSUMO3	This paper / GenScript
pGEX-4T1 SUMO3 WT	This paper / GenScript
pGEX-6P1 SUMO4 WT	This paper / GenScript
pGEX-6P1 SUMO4 M55V	This paper / GenScript

Supplemental Table 3. Antibodies

Antibody	Catalogue number	Supplier	Lot number	Concentration / Use
FLAG (Gt)	ab1257	Abcam	GR225879-35	1:1000 (WB)
FLAG M2 (Ms)	F1804	Sigma	SLCC6485	1:2000 (WB, IF)
GAPDH 6C5 (Ms)	CB1001	Calbiochem	3725987	1:5000 (WB)
HA.11 (Ms)	901501	Biolegend	B272772	1:1000 (WB, IF)
KAP1 (Ms)	NB100-47549	Novus	VK307456	1:1000 (WB)
NEDD8 (Ms)	sc-373741	Santa Cruz	J2816	1:200 (WB)
PML C7 (Ms)	ab96051	Abcam	GR3284434-1	1:1000 (IF)
PML (Rb)	PA5-79835	Invitrogen	VL31407955	1:1000 (IF)
RANGAP1 (Rb)	36067S	CST	LOT1	1:1000 (WB)
RNF4 (Goat)	AF7964	R&D	CIRA0217091	1:250 (WB)
SENP6 (Rb)	HPA024376	Merck	A106728	1:1000 (WB)
SUMO1 Y299 (Rb)	ab32058	Abcam	GR3244068-3	1:1000 (WB, IF)
SUMO1 4D12 (Rat)	MABS1223	Millipore	3540377	1:500 (WB, IF)
SUMO1 EP298 (Rb)	ab133352	Abcam	GR268526-10	1:1000 (WB, IF)
SUMO1 5B12 (Ms)	M113-3	MBL	LOT-10	1:1000 (WB, IF)
SUMO1 D11 (Ms)	sc-9060	Santa Cruz	A2021	1:200 (WB, IF)
SUMO1 852620 (Rat)	A-716	R&D Systems	02263914	1:500 (WB, IF)
SUMO1 5D8B16 (Ms)	ASM01	Cytoskeleton	LOT 3	1:1000 (WB, IF)
SUMO1 C9H1 (Rb)	4940S	CST	LOT 7	1:1000 (WB, IF)
SUMO1 21C7 (Ms)	33-2411	Invitrogen	WE326808	1:500 (WB, IF)
SUMO2/3 8A2 (Ms)	ab81371	Abcam	GR3233360-1	1:1000 (WB, IF)
SUMO2/3 EPR4602 (Rb)	ab109005	Abcam	GR154070-1	1:1000 (WB, IF)
SUMO2/3 3H12 (Rat)	ab191492	Abcam	GR274848-4	1:1000 (WB, IF)
SUMO2/3 SM23/496 (Ms)	ab190757	Abcam	GR244998-1	1:200 (WB, IF)
SUMO2/3 852908 (Rat)	A-718	R&D Systems	28464016	1:500 (WB, IF)
SUMO2/3 2H8 (Ms)	SAB1402359	Sigma	11238-2H8	1:500 (WB, IF)
SUMO2/3 12F3 (Ms)	ASM23	Cytoskeleton	LOT 11	1:1000 (WB, IF)
SUMO2/3 18H8 (Rb)	4971S	CST	LOT 7	1:1000 (WB, IF)
SUMO2/3 IE7 (Ms)	M114-3	MBL	LOT 28	1:1000 (WB, IF)
SUMO2/3 EPR300 (Rb)	ab109196	Abcam	GR96828-9	1:1000 (WB, IF)
SUMO2/3 MM0923-14V22 (Rat)	ab201594	Abcam	GR3247400-1	1:200 (WB, IF)
SUMO4 EPR7163 (Rb)	ab126606	Abcam	GR155829-1	1:1000 (WB, IF)
SUMO4 JJ085-01 (Rb)	MA5-32478	Invitrogen	WC32 16805	1:1000 (WB, IF)
SUMO4 ARC1382 (Rb)	MA535745	Life Technologies	WC3224343A	1:1000 (WB, IF)
SUMO4 IOO-19 (Rb)	M06740	Boster Biological Technologies	BS21031540	1:1000 (WB, IF)
Ubiquitin P4D1 (Ms)	sc-8017	Santa Cruz	F2320	1:200 (WB)
Donkey anti-mouse-488 AF	A21202	Invitrogen		1:2000 (IF)
Donkey anti-rabbit-488 AF	A21206	Invitrogen		1:2000 (IF)
Donkey anti-mouse-555 AF	A31570	Invitrogen		1:2000 (IF)
Donkey anti-rabbit-555 AF	A31572	Invitrogen		1:2000 (IF)
Polyclonal Swine Anti-Rabbit Immunoglobulins/HRP	P021702-2	Agilent Technologies		1:5000 (WB)
Goat anti Rat HRP	AP136P	Merck		1:5000 (WB)
Polyclonal Rabbit Anti-Mouse Immunoglobulins/HRP	P0161	Agilent Technologies		1:5000 (WB)

Supplemental Table 4. Oligonucleotides and Peptides

**Oligonucleotide**	**Sequence (5'-3') Sense – anti Sense**
siNTC	CUUACGCUGAGUACUUCGA[dT][dT]<br>[Phos]UCGAAGUACUCAGCGUAAG[dT][dT]
siSUMO1 Exon 2	CUCAAAGUCAUUGGACAGGAU[dT][dT]<br>[Phos]AUCCUGUCCAAUGACUUUGAG[dT][dT]
siSUMO1 UTR	CCUUCAUAUUACCCUCUCCUU[dT][dT]<br>[Phos]AAGGAGAGGGUAAUAUGAAGG[dT][dT]
siSUMO2 UTR A	GUACGUAGCUGUUACAUGU[dT][dT]<br>[Phos]ACAUGUAACAGCUACGUAC[dT][dT]
siSUMO2 UTR B	GCGUCUUGUUGUUUAAAUA[dT][dT]<br>[Phos]UAUUUAAACAACAAGACGC[dT][dT]
siSUMO3 Exon 2	GCAAGCUGAUGAAGGCCUA[dT][dT]<br>[Phos]UAGGCCUUCAUCAGCUUGC[dT][dT]
siSUMO3 UTR	GGGAUGAAUCUGUAACUUA[dT][dT]<br>[Phos]UAAGUUACAGAUUCAUCCC[dT][dT]
siSUMO4 A	ACCUCCCGUAGGCUGUUGAAA[dT][dT]<br>[Phos]UUUCAACAGCCUACGGGAAGGU[dT][dT]
siSUMO4 B	ACUGUGCAGGUUUGUCUGUUC[dT][dT]<br>[Phos]GAACAGACAAACCUGCACAGU[dT][dT]
siSENP6	CACAGGAUUAACAACCAAGAA[dT][dT]<br>[Phos]UUCUUGGUUGUUAAUCCUGUG[dT][dT]
siRNF4 Exon 11	[Phos]AACGAUGAGACGUCCAUUC[dT][dT]<br>GAAUGGACGUCUCAUCGUU[dT][dT]
siRNF4 Exon 9	[Phos]UCACAUAUACGUCUCUGUC[dT][dT]<br>GACAGAGACGUAUAUGUGA[dT][dT]
**Peptides**	
SUMO1 # 1	MSDQEAKPSTEDLGDKKEGE
SUMO1 # 2	EGEYIKLKVIGQDSSEIHFK
SUMO1 # 3	HFKVKMTTHLKKLKESYCQR
SUMO1 # 4	CQRQGVPMNSLRFLFEGQRI
SUMO1 # 5	QRIADNHTPKELGMEEEDVI
SUMO1 # 6	DVIEVYQEQTGGHSTV
SUMO2 # 1	MADEKPKEGVKTENNDHINL
SUMO2 # 2	INLKVAGQDGSVVQFKIKRH
SUMO2 # 3	KRHTPLSKLMKAYCERQGLS
SUMO2 # 4	GLSMRQIRFRFDGQPINETD
SUMO2 # 5	ETDTPAQLEMEDEDTIDVFQQQTGGVY
SUMO4 # 1	MANEKPTEEVKTENNNHINL
SUMO4 # 2	INLKVAGQDGSVVQFKIKRQ
SUMO4 # 3	KRQTPLSKLMKAYCEPRGLS
SUMO4 # 4	GLSMKQIRFRFGGQPISGTD
SUMO4 # 5	GTDKPAQLEMEDEDTIDVFQQPTGGVY

Supplemental Table 5. Cell lines

Cell line / Supplier	Growth conditions	RRID
U2OS FlpIn (Grant Stewarts, University of Birmingham)	DMEM, 10% FBS, 1% Penicillin/Streptomycin	N/A
U2OS FlpIn 6xHis-FLAG SUMO1 WT (This study)	DMEM, 10% FBS, 1% Penicillin/Streptomycin, 100 mg / mL Hygromycin B	N/A
U2OS FlpIn 6xHis-FLAG SUMO2 WT (This study)	DMEM, 10% FBS, 1% Penicillin/Streptomycin, 100 mg / mL Hygromycin B	N/A
U2OS FlpIn 6xHis-FLAG SUMO3 WT (This study)	DMEM, 10% FBS, 1% Penicillin/Streptomycin, 100 mg / mL Hygromycin B	N/A
U2OS FlpIn HA-FLAG SUMO4 WT (This study)	DMEM, 10% FBS, 1% Penicillin/Streptomycin, 100 mg / mL Hygromycin B	N/A
A427 (ATCC)	EMEM, 10% FBS, 1% Penicillin/Streptomycin, 1% NEAA, 1mM Sodium Pyruvate	CVCL_1055
CALU6 (ATCC)	EMEM, 10% FBS, 1% Penicillin/Streptomycin, 1% NEAA, 1mM Sodium Pyruvate	CVCL_0236
CAL51 (ATCC)	DMEM, 10% FBS, 1% Penicillin/Streptomycin	CVCL_1110
HCT116 (ATCC)	DMEM, 10% FBS, 1% Penicillin/Streptomycin	CVCL_0291
HEK293 TREx FlpIn (Invitrogen)	DMEM, 10% FBS, 1% Penicillin/Streptomycin	CVCL_U427

Supplemental Table 6. Chemicals

Chemical / Treatment	Manufacturer / Product code	Dosage / Time
Camptothecin (CPT)	Merck 208925	1 μM 3 hr
Hydroxyurea (HU)	Merck H8627	5 mM 3 hr
Hydrogen Peroxide (H_2O_2)	Merck 31642	125 μM 15 min
Lipopolysaccharide (LPS)	Merck L4391	1000 ng/mL 3 hr
MG132	Merck 474787	5 μg/ mL 3 hr
CB-5083	Selleck S8101-SEL	100 nM 3 hr
17-AAG	Cambridge Biosciences 11039-1mg-CAY	5 μM 3 hr
Sorbitol	Merck S1876	500 mM 0.5 hr
ICRF-193	Merck I4659-1MG	100 μM 1 hr
Cobalt Chloride ($CoCl_2$)	SLS CHE1618	200 mM 3 hr
Doxycycline	Merck D9891	1 μg/ mL 72 hr
Hygromycin B	Invitrogen H044-81VS	100 μg/ mL
Iodoacetamide (IAA)	Merck I1149	200 μM
Ionising Radiation	CellRad Irradiator	4 Gy
ML-792	Selleck S8697	10 μM 6 hr
TAK-243	Selleck S8341	10 μM 6 hr
MLN4924	Selleck S7109	10 μM 6 hr

Uncropped membranes

Figure 1b

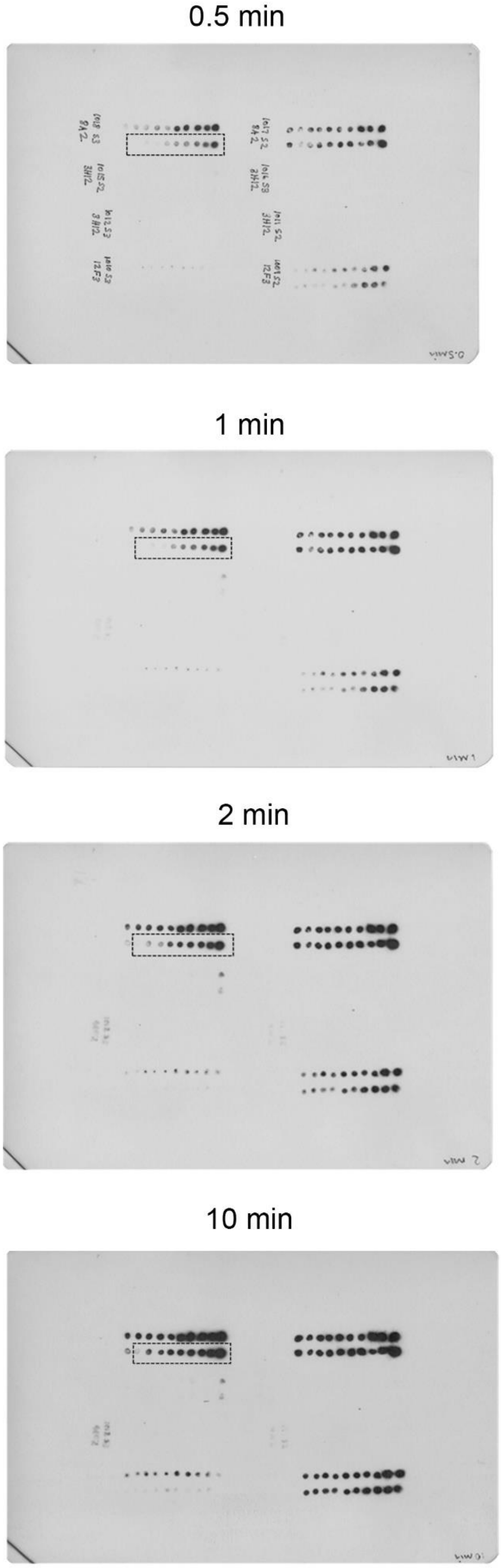

Figure 2a

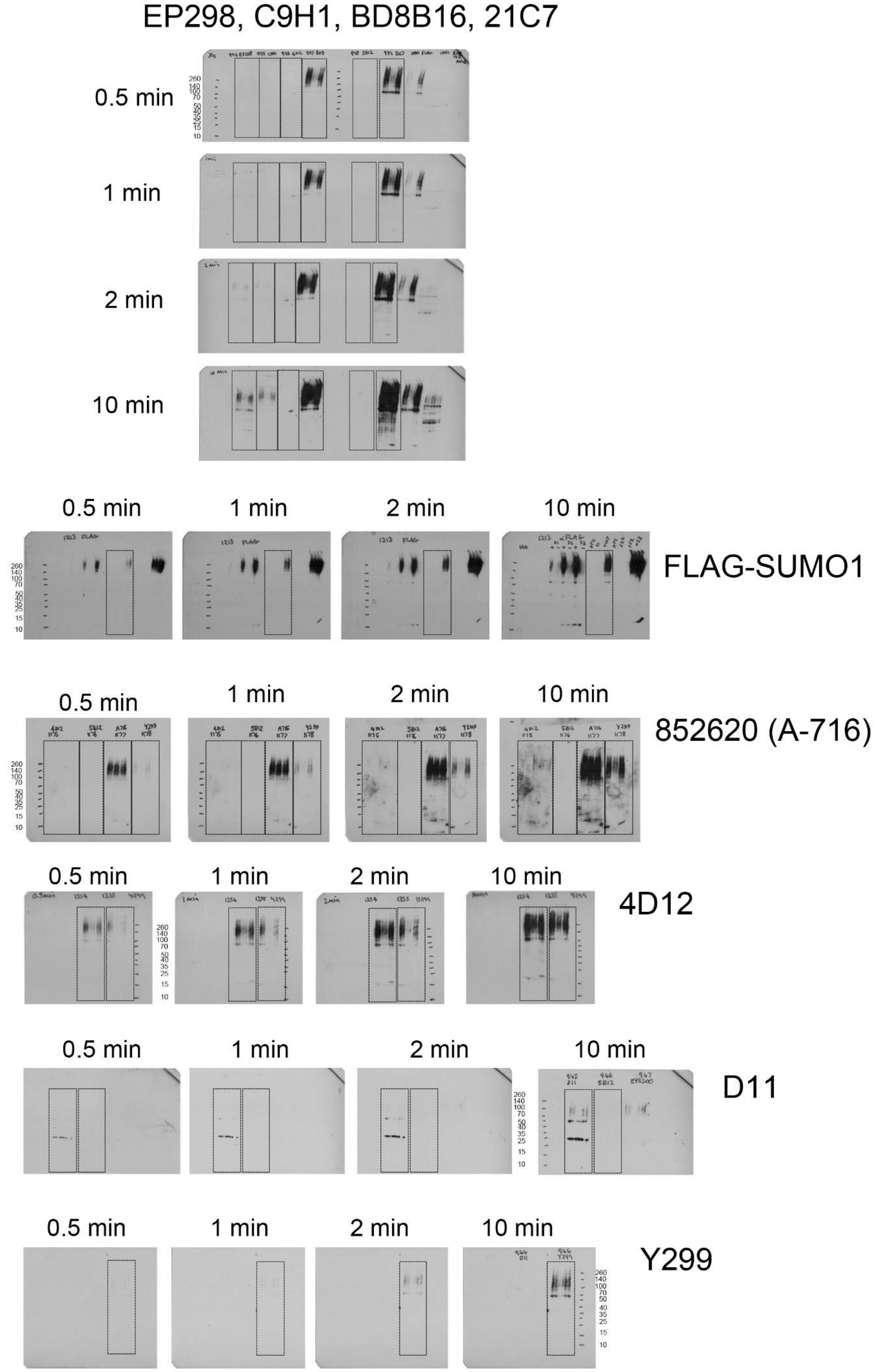
EP298, C9H1, BD8B16, 21C7
0.5 min
1 min
2 min
10 min
0.5 min
1 min
2 min
10 min
FLAG-SUMO1
0.5 min
1 min
2 min
10 min
852620 (A-716)
0.5 min
1 min
2 min
10 min
4D12
0.5 min
1 min
2 min
10 min
D11
0.5 min
1 min
2 min
10 min
Y299

Figure 2c

FLAG SUMO2 and FLAG SUMO3

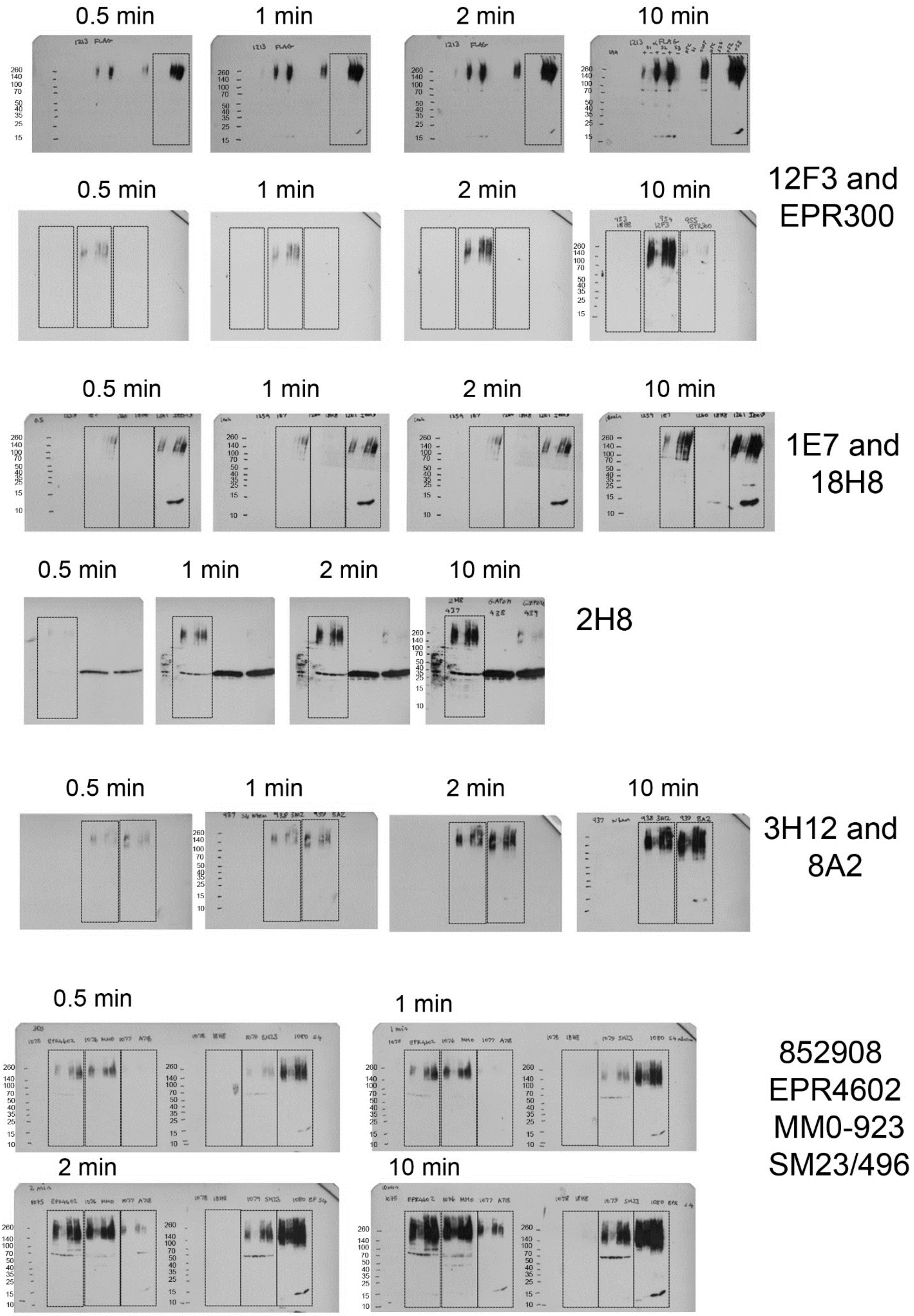

Figure 2e

EPR7163

0.5 min

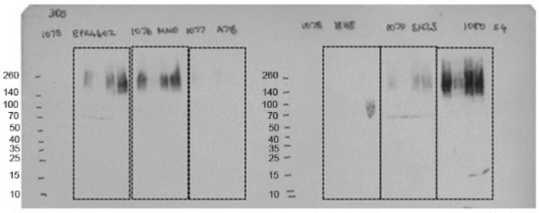

1 min

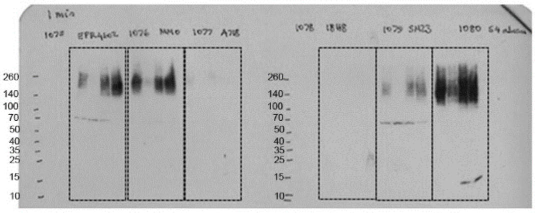

2 min

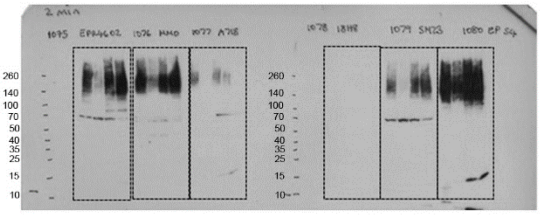

10 min

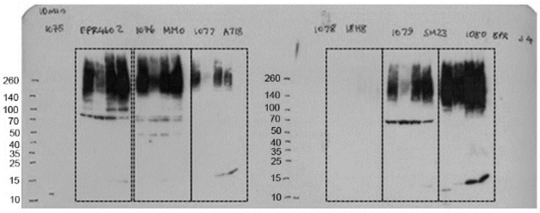

ARC1382 and JJ-085

0.5 min

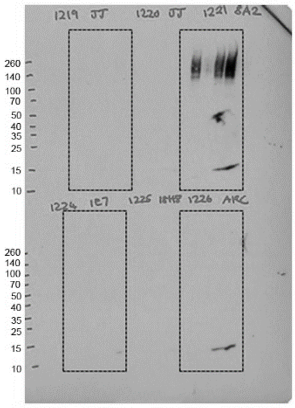

1 min

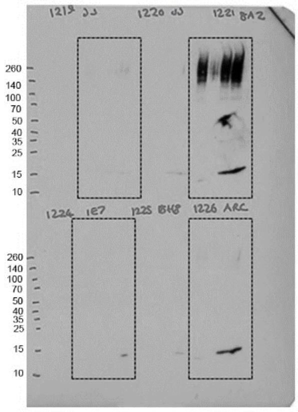

2 min

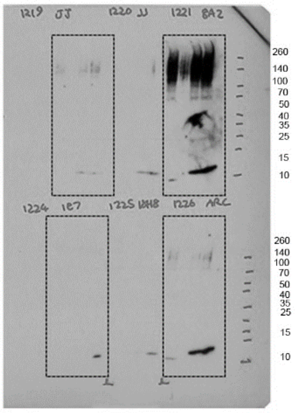

10 min

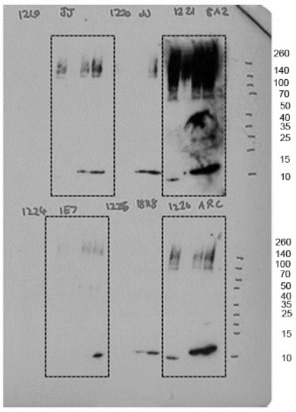

IOO-19

0.5 min

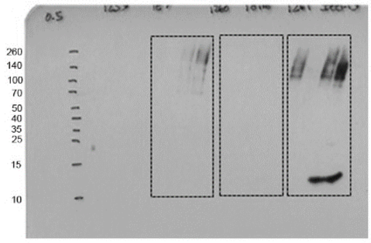

1 min

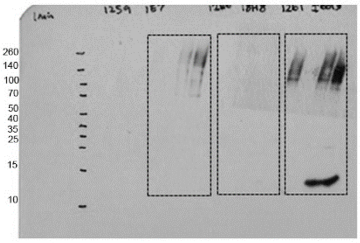

2 min

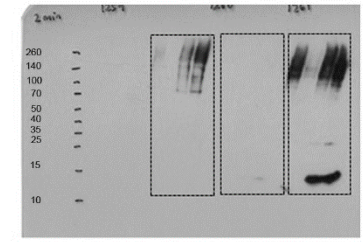

10 min

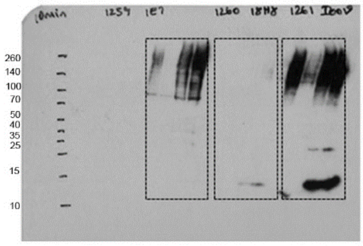

Figure 3b

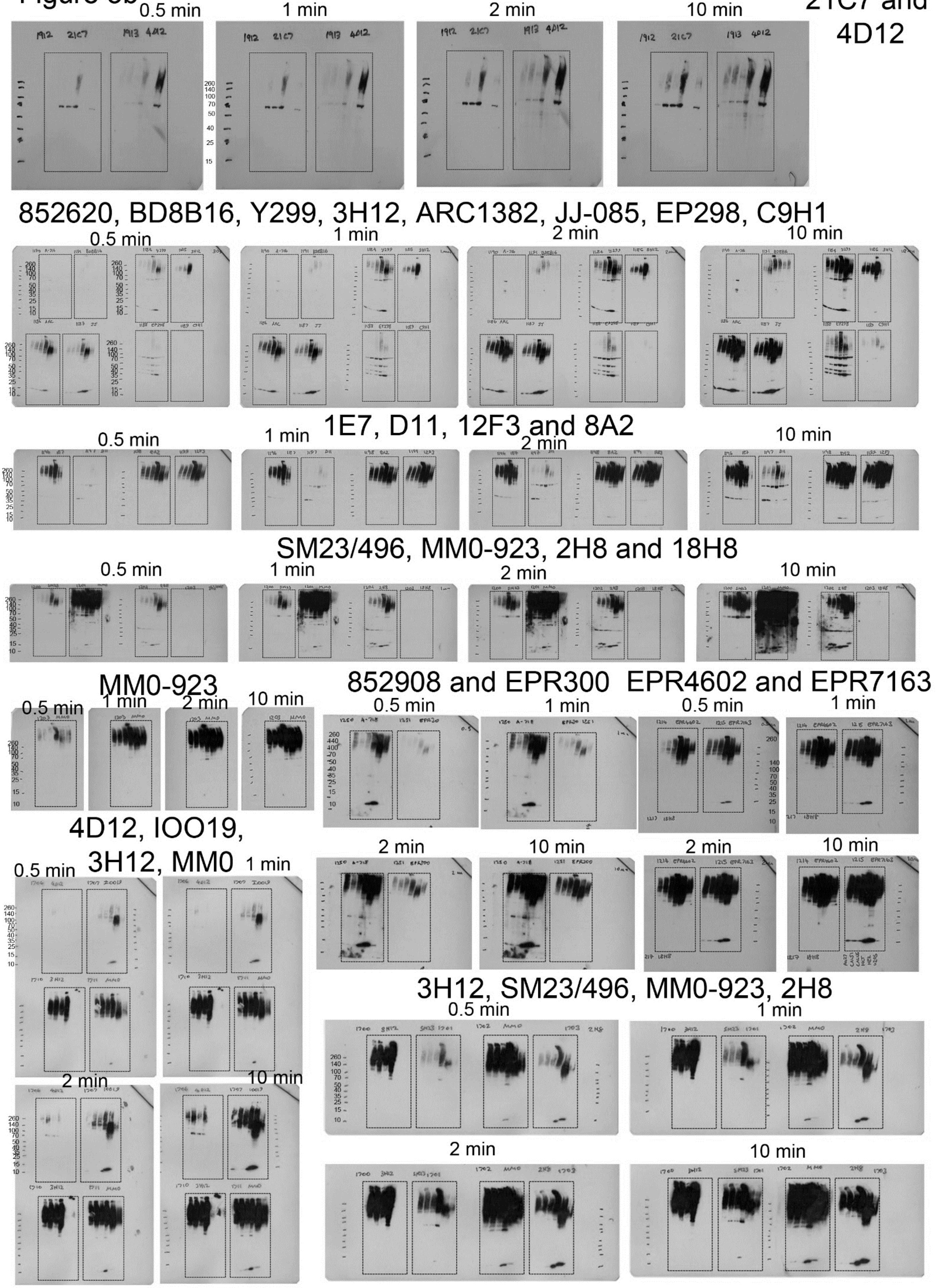
0.5 min
1 min
2 min
10 min
21C7 and 4D12
852620, BD8B16, Y299, 3H12, ARC1382, JJ-085, EP298, C9H1
1E7, D11, 12F3 and 8A2
SM23/496, MM0-923, 2H8 and 18H8
MM0-923
852908 and EPR300
EPR4602 and EPR7163
4D12, IOO19, 3H12, MM0
3H12, SM23/496, MM0-923, 2H8
260
140
100
70
50
40
35
25
15
10

Figure 3c

ARC1382, EPR7163, IOO-19, JJ-085

5 s

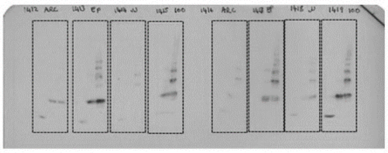

10 s

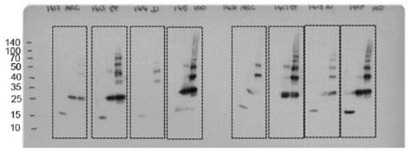

15 s

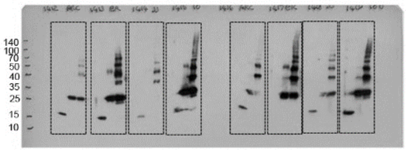

30 s

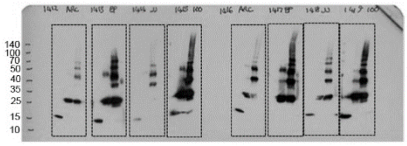

1 min

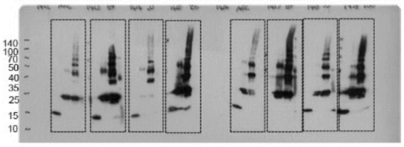

2 min

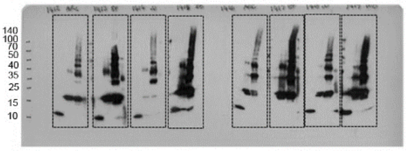

5 min

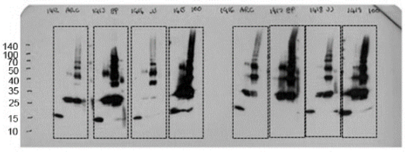

10 min

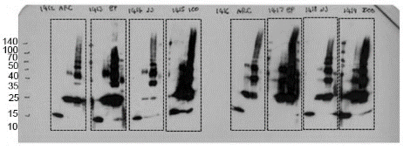

12F3. 1E7, 2H8, 3H12 8A2, 18H8, 852908, EPR300, EPR4602, MM0, SM23

10 s

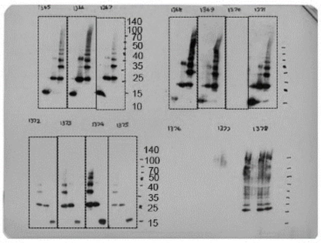

15 s

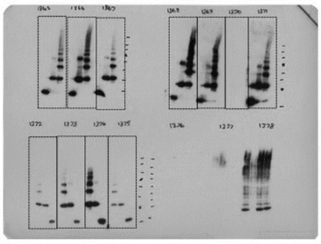

30 s

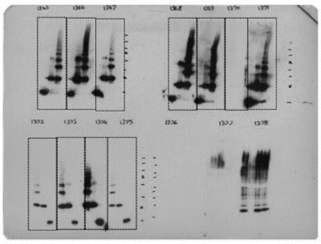

1 min

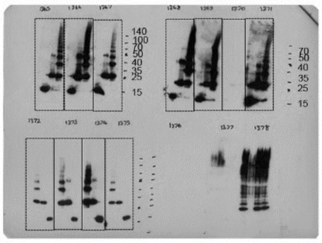

2 min

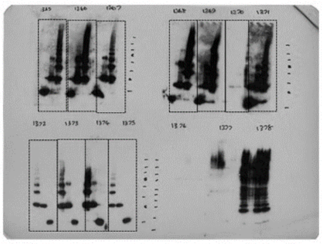

5 min

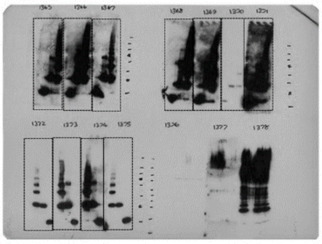

10 min

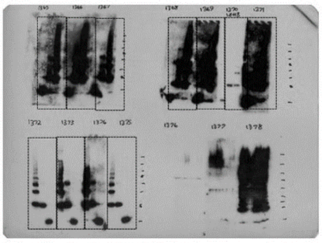

12F3, 1E7, 2H8, 3H12, 8A2, 18H8. 852908, EPR300, EPR4602, MM0, SM23, ARC1382, JJ-085, IOO-19, 852620

5 s

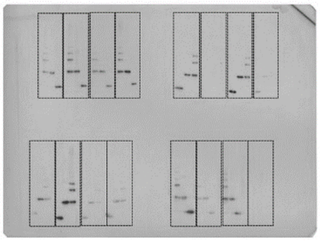

10 s

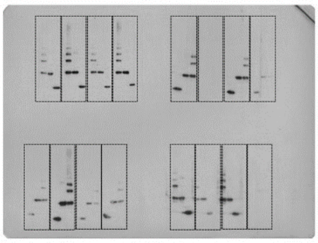

30 s

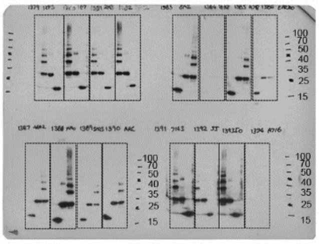

1 min

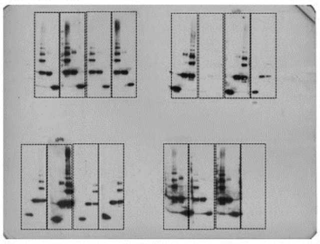

2 min

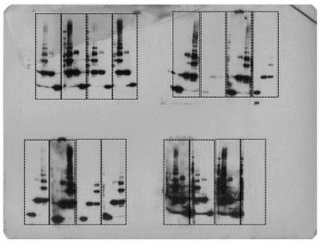

5 min

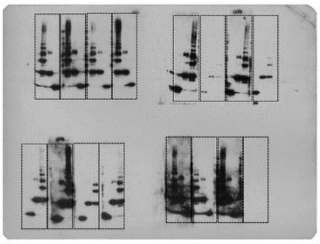

10 min

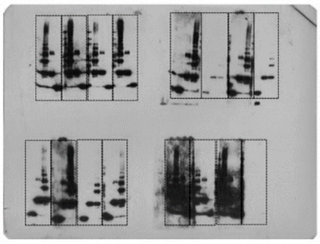

Figure 3e

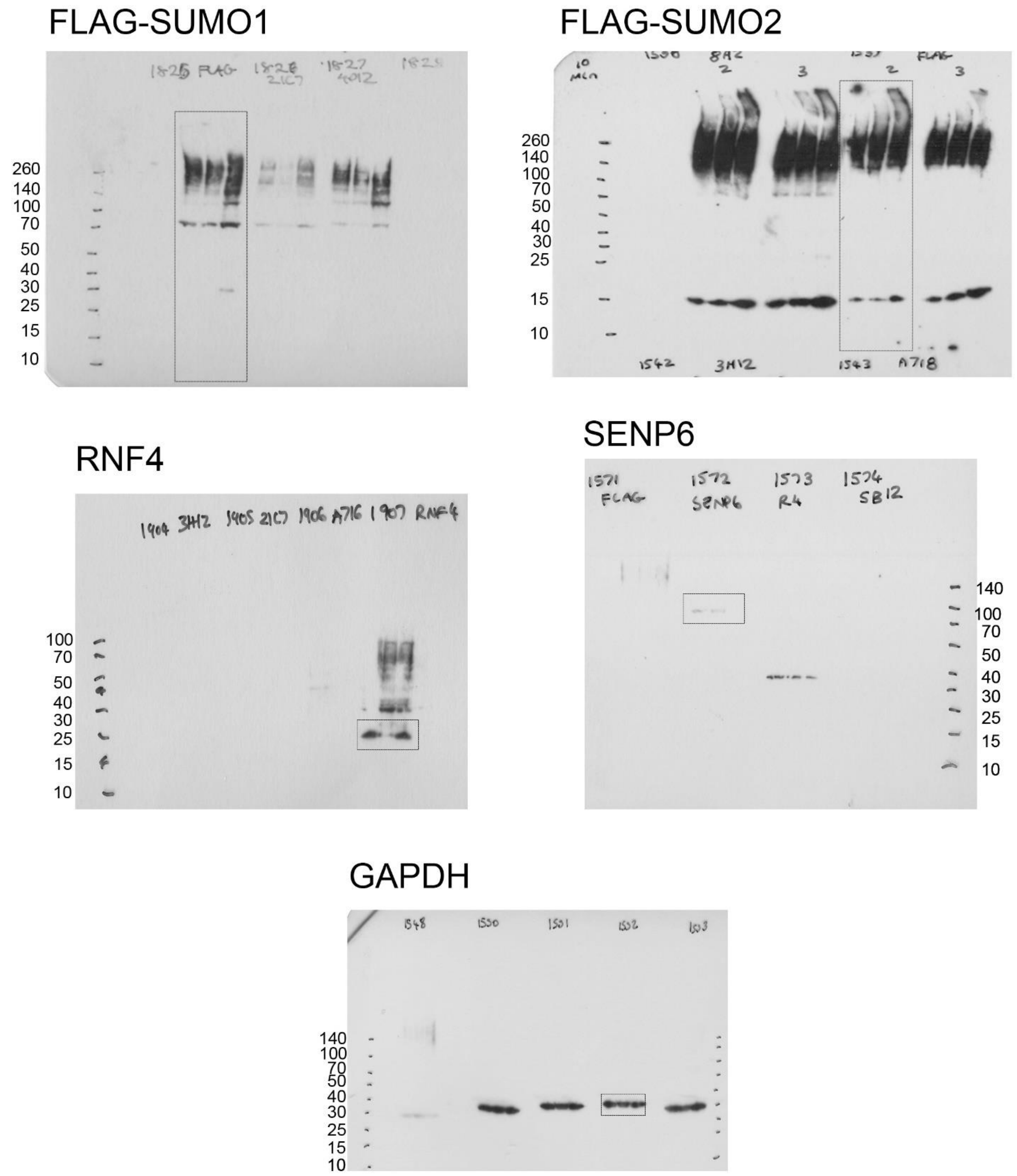
FLAG-SUMO1
260
140
100
70
50
40
30
25
15
10
FLAG-SUMO2
260
140
100
70
50
40
30
25
15
10
RNF4
100
70
50
40
30
25
15
10
SENP6
140
100
70
50
40
30
25
15
10
GAPDH
140
100
70
50
40
30
25
15
10

Figure 3f

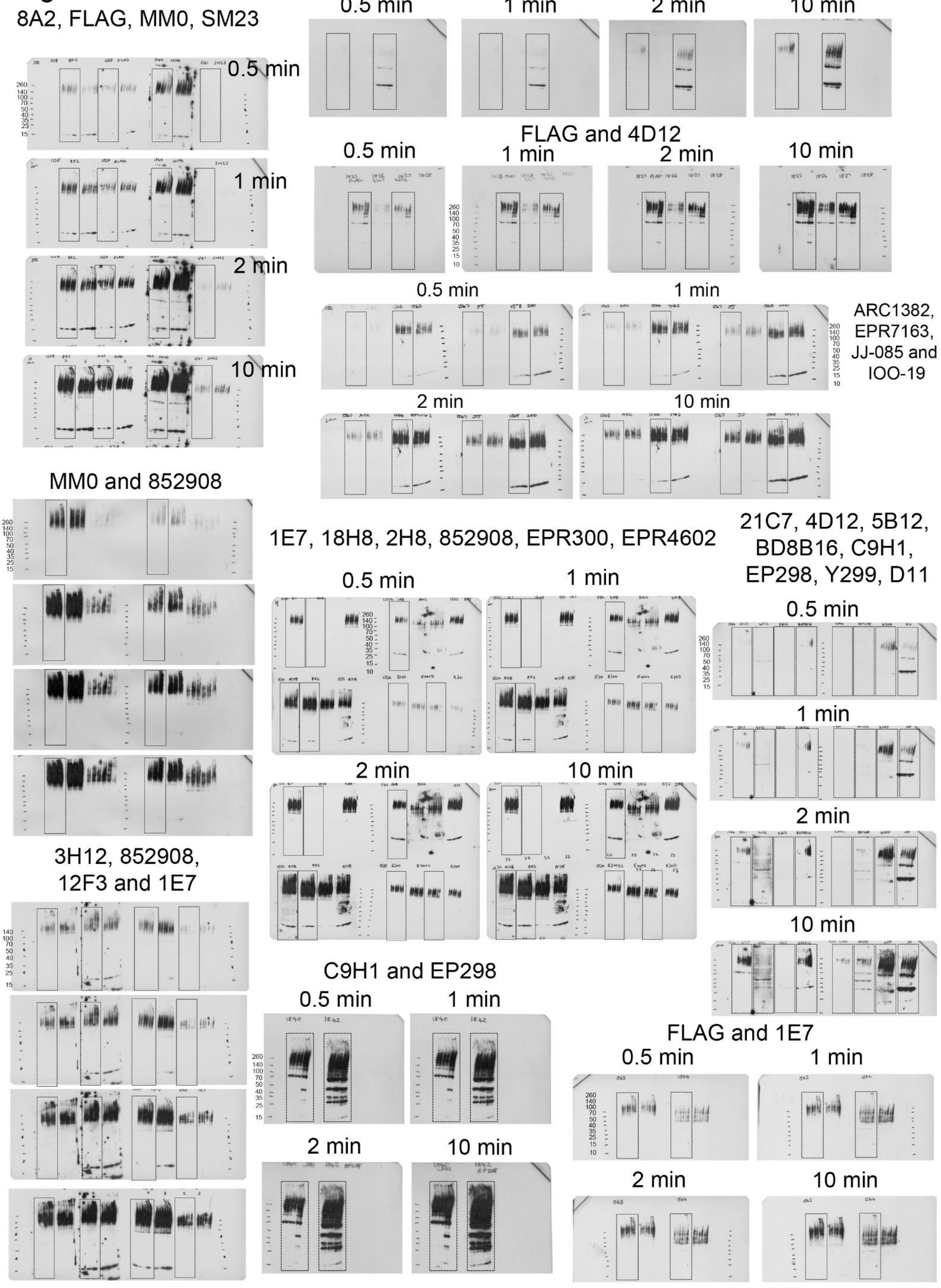
8A2, FLAG, MM0, SM23
0.5 min
1 min
2 min
10 min
BD8B16 and D11
0.5 min
1 min
2 min
10 min
FLAG and 4D12
0.5 min
1 min
2 min
10 min
ARC1382, EPR7163, JJ-085 and IOO-19
0.5 min
1 min
2 min
10 min
MM0 and 852908
1E7, 18H8, 2H8, 852908, EPR300, EPR4602
0.5 min
1 min
2 min
10 min
21C7, 4D12, 5B12, BD8B16, C9H1, EP298, Y299, D11
0.5 min
1 min
2 min
10 min
3H12, 852908, 12F3 and 1E7
C9H1 and EP298
0.5 min
1 min
2 min
10 min
FLAG and 1E7
0.5 min
1 min
2 min
10 min
260
140
100
70
50
40
35
25
15
10

Figure 4a

GAPDH

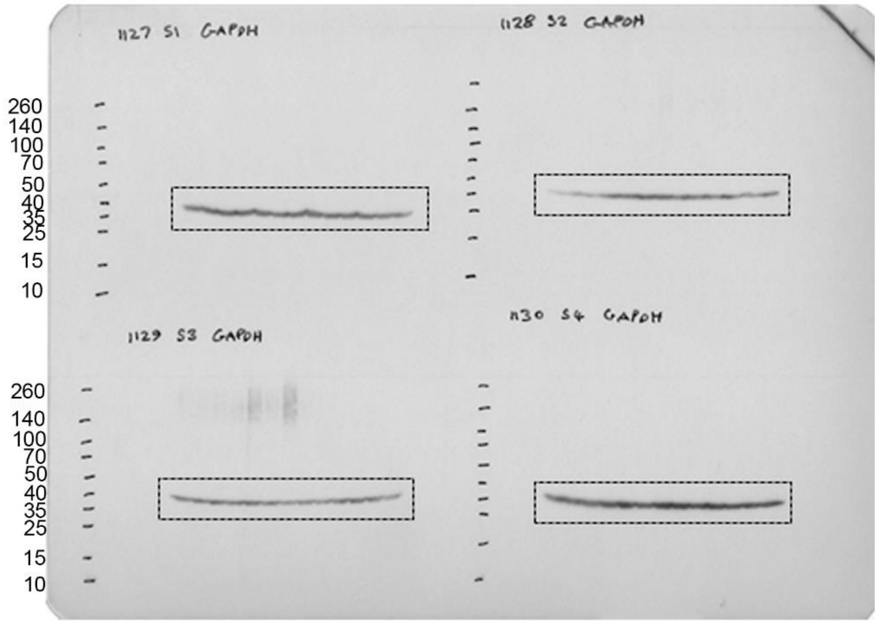

FLAG SUMO1

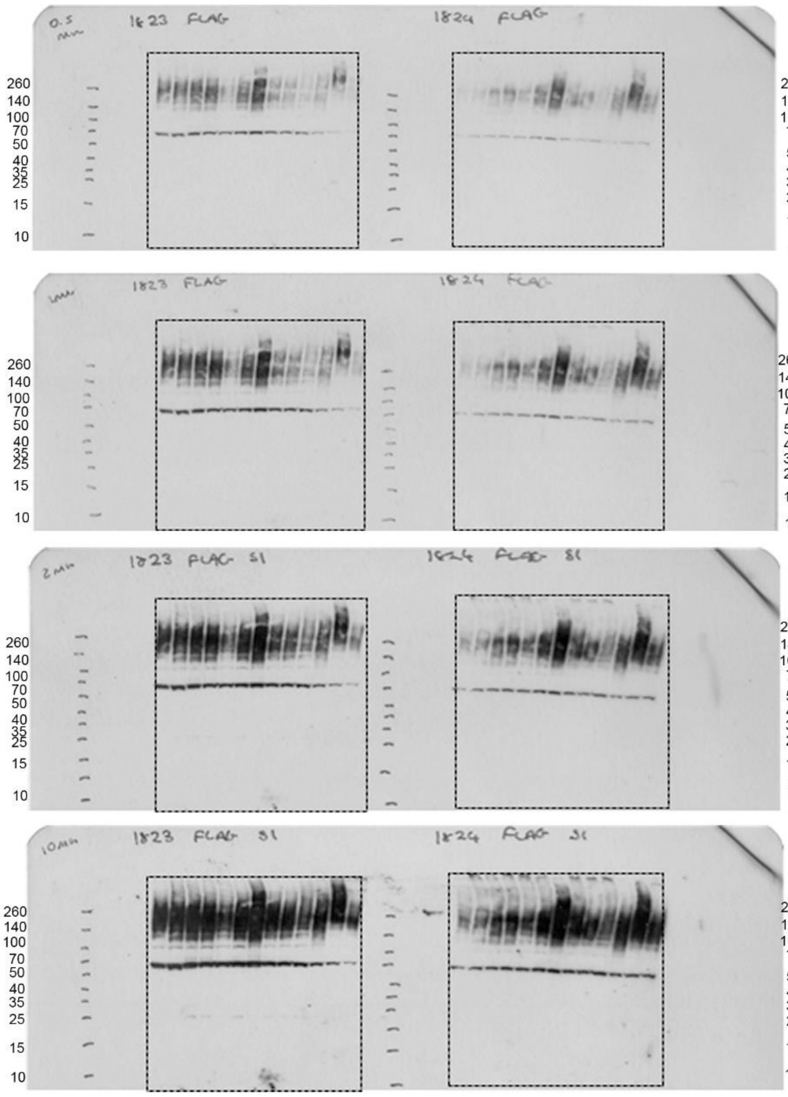

FLAG SUMO2

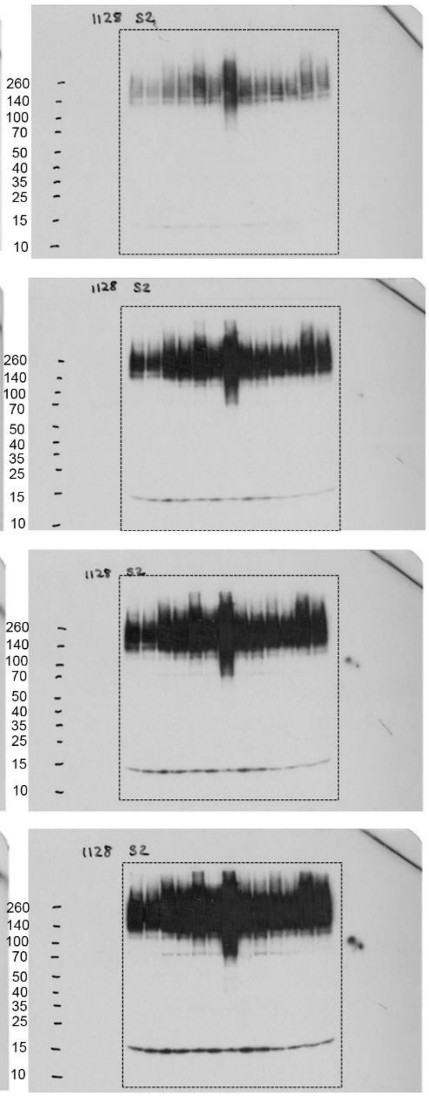

Figure 4b

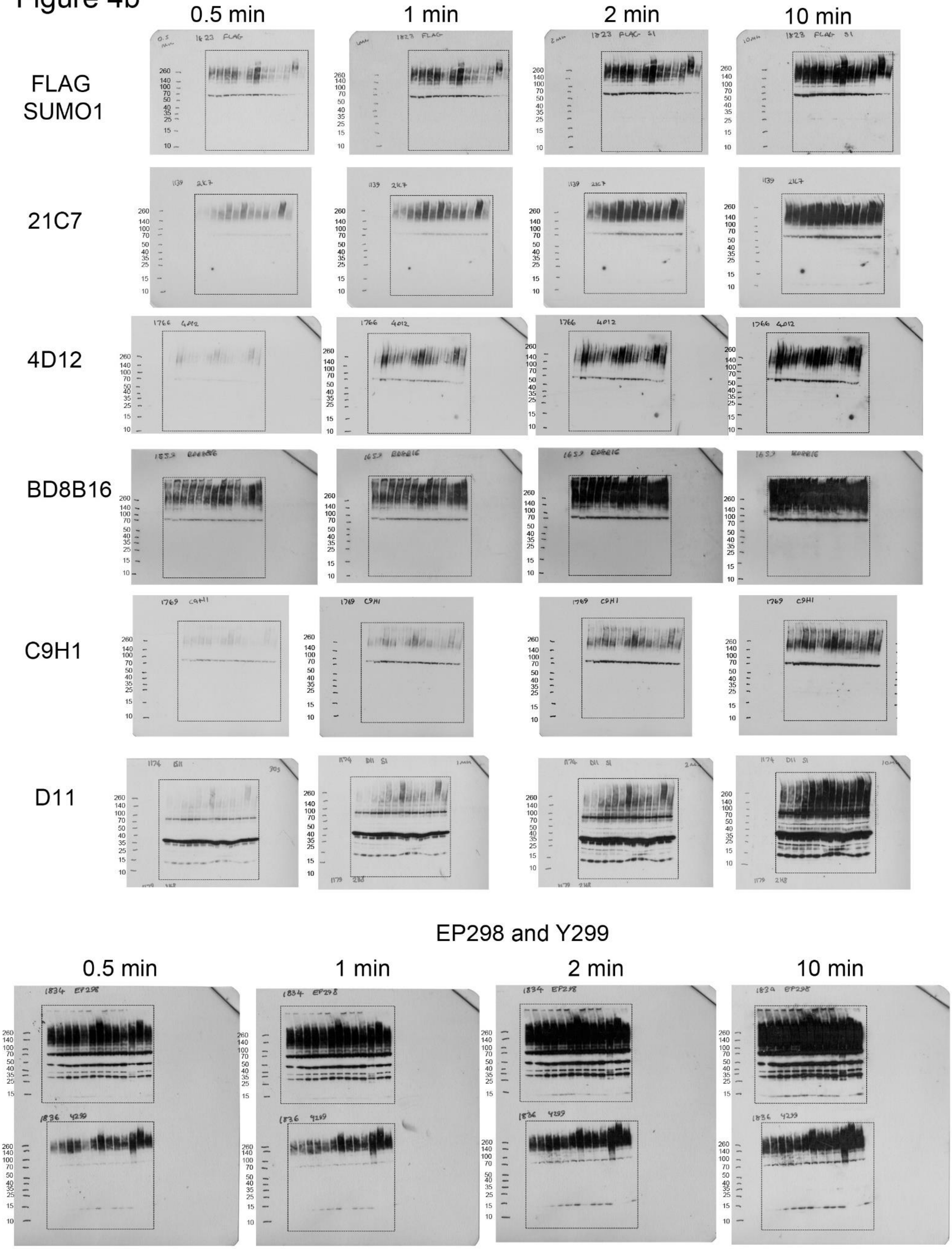
0.5 min
1 min
2 min
10 min
FLAG SUMO1
21C7
4D12
BD8B16
C9H1
D11
260
140
100
70
50
40
35
25
15
10
EP298 and Y299

Figure 4b

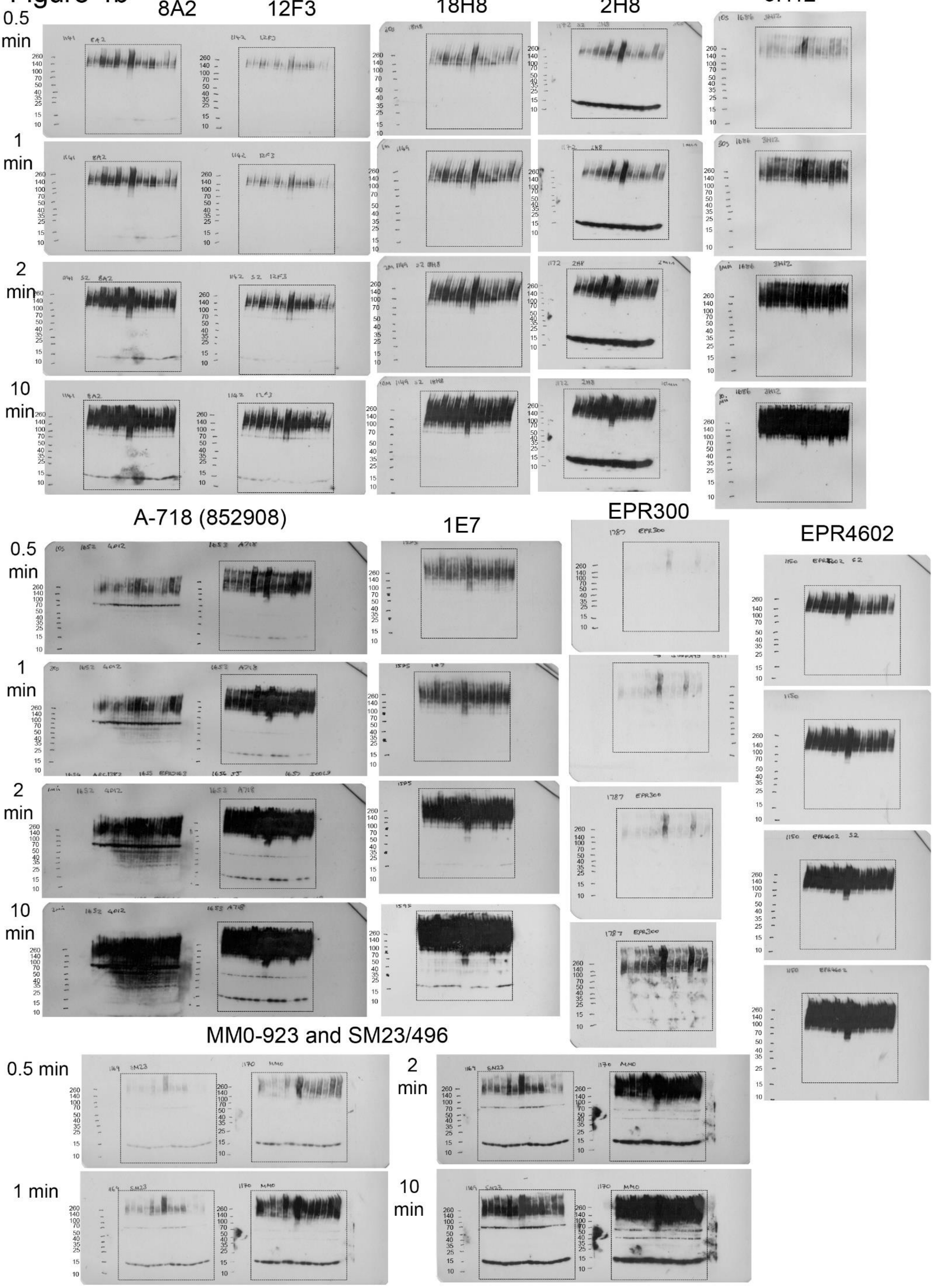
8A2
12F3
18H8
2H8
3H12
0.5 min
1 min
2 min
10 min
260 140 100 70 50 40 35 25 15 10
A-718 (852908)
1E7
EPR300
EPR4602
MM0-923 and SM23/496

Figure 4d

FLAG-HA SUMO4 and ARC1382

0.5 min

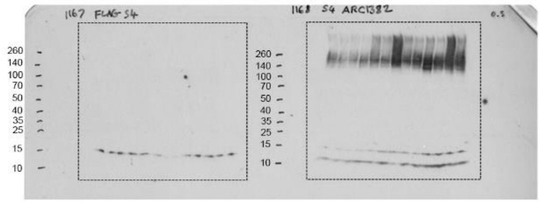

1 min

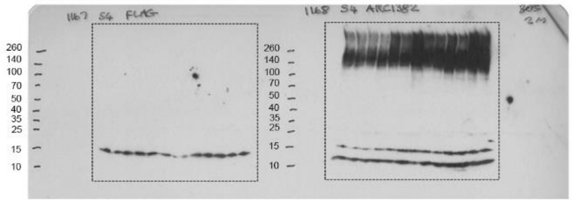

2 min

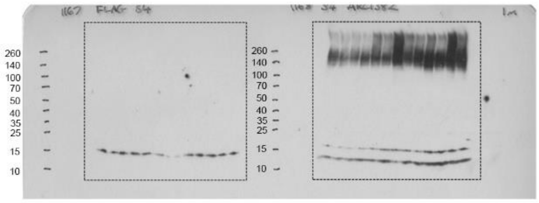

10 min

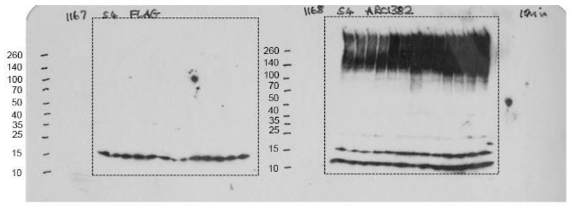

EPR7163

0.5 min

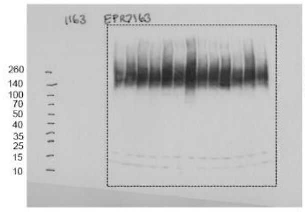

1 min

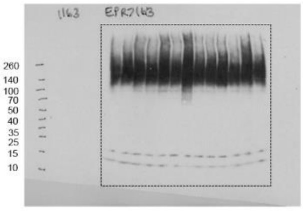

2 min

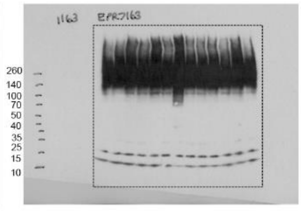

10 min

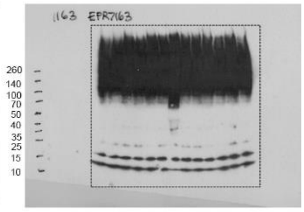

IOO-19

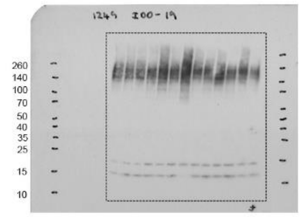

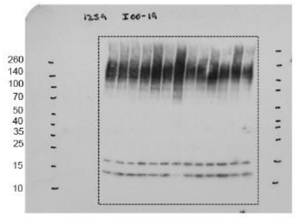

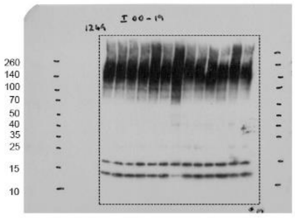

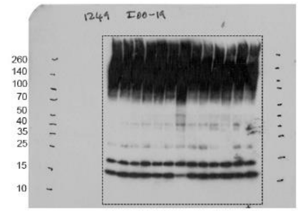

JJ-085

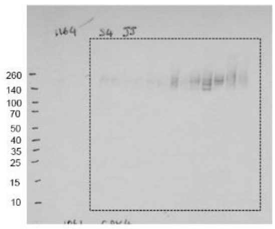

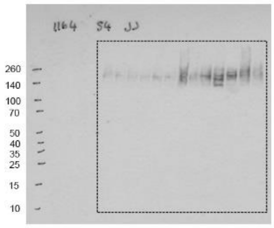

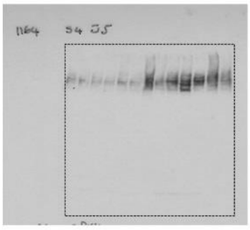

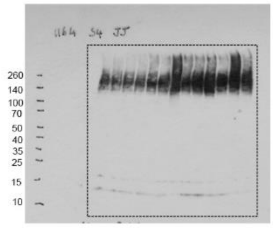

Figure 6

6a

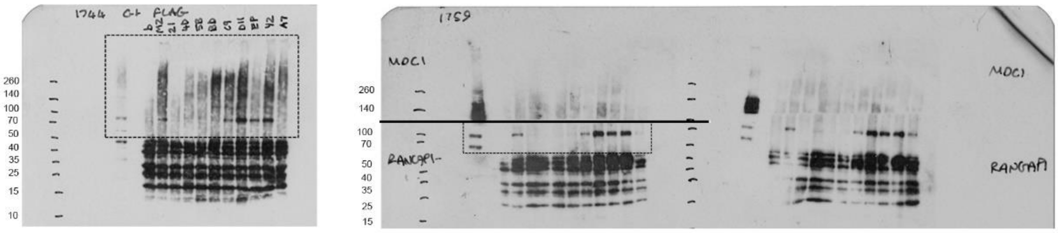

6d

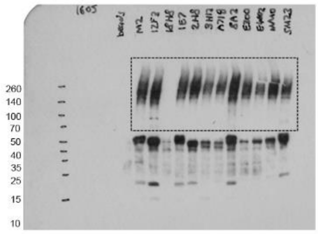

6f

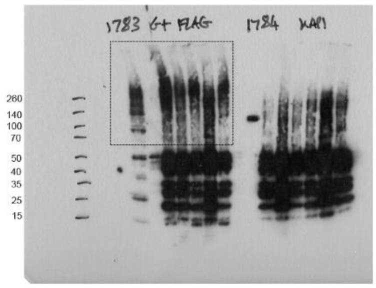

6g

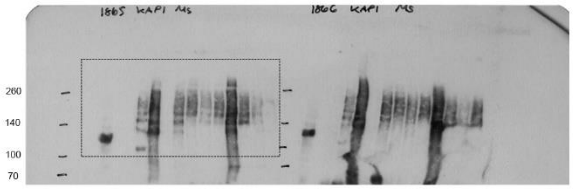

Supplemental Figure 2

2b

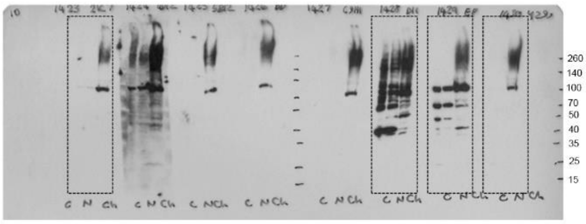

2c

Y299 852620 NEDD8

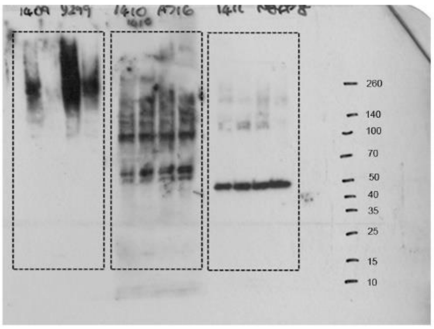

Ubiquitin

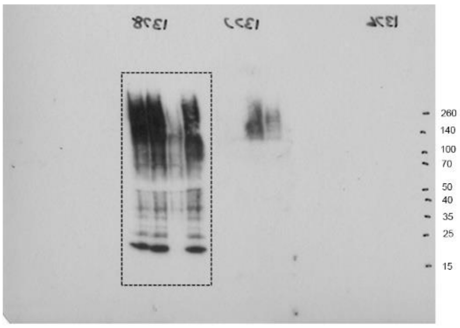

GAPDH

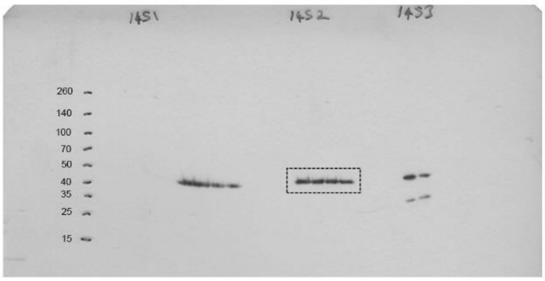

Supplemental Figure 2

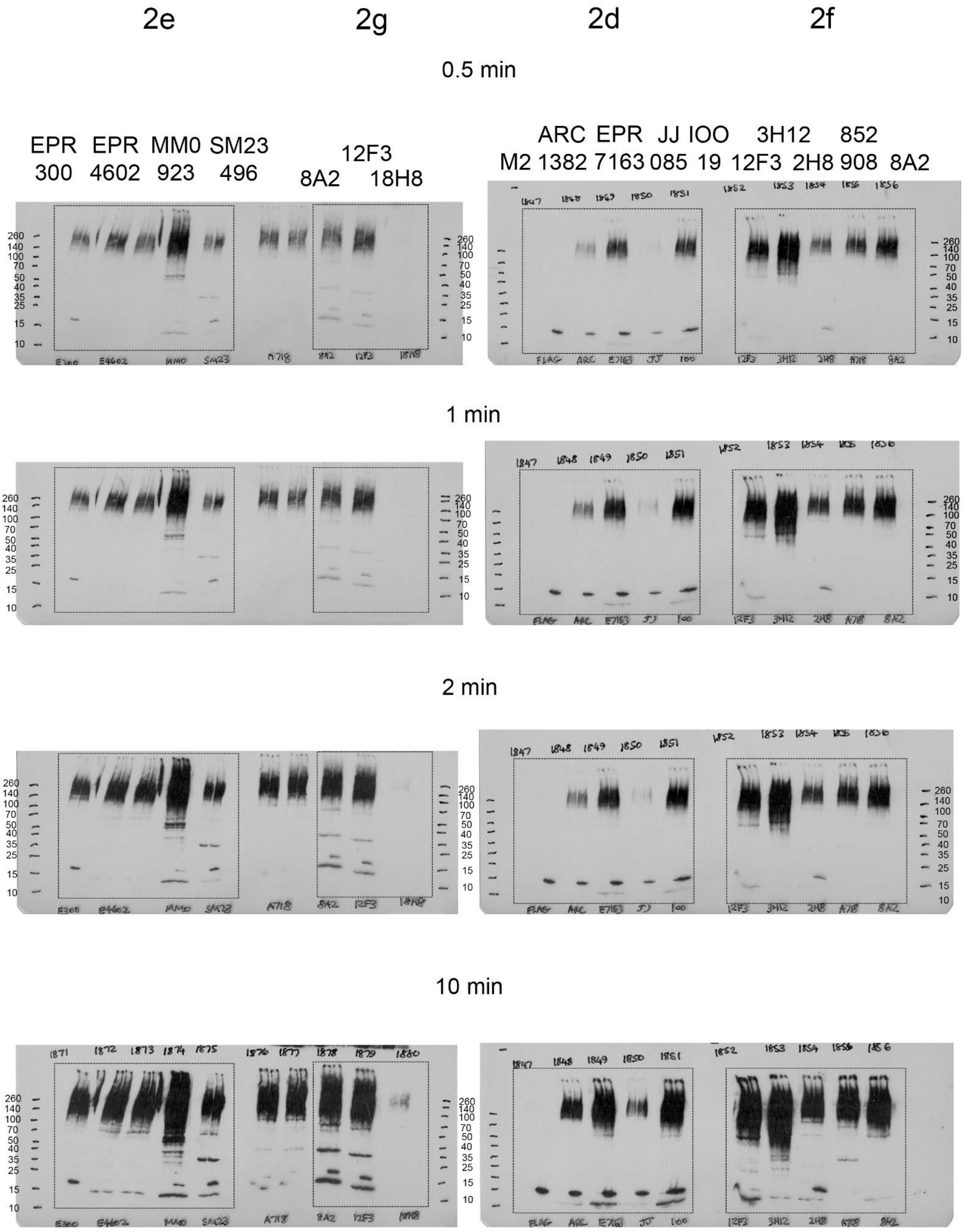

Representative immunoblots to demonstrate lack of SUMO1 detection by MAb 5B12.

Related to figure 2a - parallel membranes from the same lysates, gel and transfer were cut and probed with 5B12, D11, FLAG or Y299 MAbs.

Related to figure 4b 5B12 failed to detect changes in SUMO1 conjugates after stress. Note the signal from the same lysates probed in parallel with D11.

5B12 WB

Related to Figure 2a

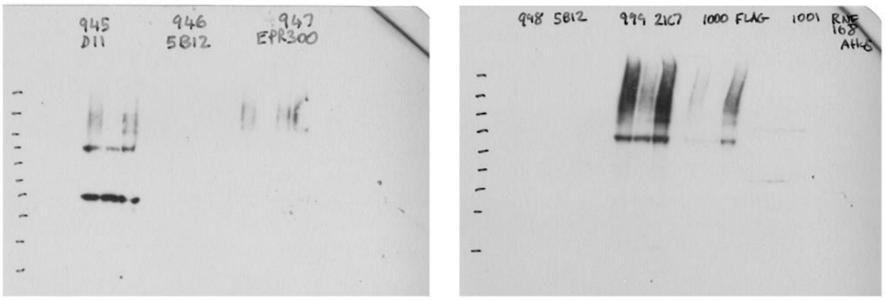

Related to Figure 4b

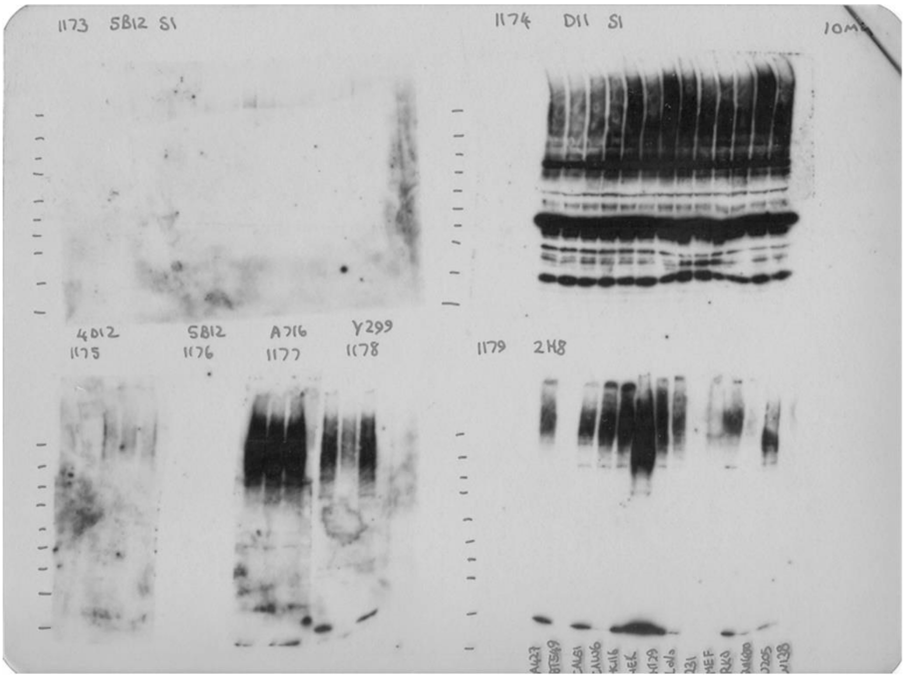

Supplement: Supplementary file 1 — Supplementary Information. [file 41598_2022_25665_MOESM1_ESM.pdf]
